# Comparison of Goto-Kakizaki rats and high fat diet-induced obese rats: Are they reliable models to study Type 2 *Diabetes mellitus*?

**DOI:** 10.1371/journal.pone.0189622

**Published:** 2017-12-08

**Authors:** Wilson Mitsuo Tatagiba Kuwabara, Ana Carolina Panveloski-Costa, Caroline Naomi Fukusawa Yokota, Joice Naiara Bertaglia Pereira, Jorge Mancini Filho, Rosangela Pavan Torres, Sandro Massao Hirabara, Rui Curi, Tatiana Carolina Alba-Loureiro

**Affiliations:** 1 Department of Physiology and Biophysics, Institute of Biomedical Sciences, University of São Paulo, São Paulo, Brazil; 2 Cruzeiro do Sul University, São Paulo, Brazil; 3 Faculty of Pharmaceutical Sciences, University of São Paulo, São Paulo, Brazil; Max Delbruck Centrum fur Molekulare Medizin Berlin Buch, GERMANY

## Abstract

Type 2 Diabetes mellitus (T2DM) is an evident growing disease that affects different cultures throughout the world. T2DM occurs under the influence of three main factors: the genetic background, environmental and behavioral components. Obesity is strongly associated to the development of T2DM in the occident, while in the orient most of the diabetic patients are considered lean. Genetics may be a key factor in the development of T2DM in societies where obesity is not a recurrent public health problem. Herein, two different models of rats were used to understand their differences and reliability as experimental models to study the pathophysiology of T2DM, in two different approaches: the genetic (GK rats) and the environmental (HFD-induced obese rats) influences. GK rats were resistant to weight gain even though food/energy consumption (relative to body weight) was higher in this group. HFD, on the other hand, induced obesity in Wistar rats. White adipose tissue (WAT) expansion in this group was accompanied by immune cells infiltration, inflammation and insulin resistance. GK rats also presented WAT inflammation and insulin resistance; however, no immune cells infiltration was observed in the WAT of this group. Liver of HFD group presented fat accumulation without differences in inflammatory cytokines content, while liver of GK rats didn’t present fat accumulation, but showed an increase of IL-6 and IL-10 content and glycogen. Also, GK rats showed increased plasma GOT and GPT. Soleus muscle of HFD presented normal insulin signaling, contrary to GK rats, which presented higher content of basal phosphorylation of GSK-3β. Our results demonstrated that HFD developed a mild insulin resistance in Wistar rats, but was not sufficient to develop T2DM. In contrast, GK rats presented all the typical hallmarks of T2DM, such as insulin resistance, defective insulin production, fasting hyperglycemia/hyperinsulinemia and lipid plasma alteration. Thus, on the given time point of this study, we may conclude that only GK rats shown to be a reliable model to study T2DM.

## Introduction

By 2030, *diabetes mellitus* (DM) will be the 7^th^ leading cause of death worldwide, staying behind only of ischemic heart diseases, cerebral disease, HIV/AIDS, Chronic Obstructive Pulmonary Disease (COPD), lower respiratory infections, and trachea, bronchus and lung cancer [1]. The incidence of DM has risen vertiginously and, in 2014, as claimed by the World Health Organization (WHO), reached the hallmark of 422 million individuals [2]. This augmentation is mainly because of unhealthy dietary habits, like increased intake of sugar, fats, processed foods, and sweetened beverages, related to low consumption of fruits and vegetables, as well as sedentary lifestyle. DM is associated with complications that affect patient’s quality of life, for example, more than 50% of diabetic patients present other physiological disorder, such as cardiovascular diseases (heart attacks and strokes) [3], higher susceptibility to infections [4], kidney failure [5], and retinopathy [6].

DM is a syndrome characterized by carbohydrate, lipid, and protein metabolism disorders and it occurs as a result either from deficiency/absence of insulin secretion or resistance to the action of this hormone. Type 2 DM (T2DM) is the most common type of DM and it is characterized by insulin resistance in skeletal muscle, adipose tissue, and liver. Defective β-cell secretory function, fasting hyperglycemia and hyperinsulinemia and increased hepatic glucose production are also hallmarks of T2DM [7]. Development of T2DM is due to a combination of three different factors: genetic, environmental and behavioral [8]. In this study, we aim to compare two of the components that interfere in T2DM development: genetic *versus* environmental (diet) factors.

Goto Kakizaki (GK) rat, a non-obese and spontaneous (genetic) T2DM experimental model, has been widely used to investigate the development of T2DM and its complications [9–14]. These animals were obtained by repetition of selective breeding of glucose intolerant Wistar rats [15]. Males and females rats are similarly affected by the diabetic condition and differences are observed even before birth. In uterus, GK fetus shows reduced pancreatic β-cell mass; however, right after birth, rats present normal blood glucose. When GK rats are 28 days of age, basal hyperglycemia, impaired insulin secretion by pancreatic β-cells and increased hepatic glucose production are observed [16–18]. Only after 56 days of life, GK rats develop peripheral insulin resistance [19]. Considering that the genetic factor contributes to etiology and progression of T2DM, various studies have been developed to identify susceptible genes in GK model. Recently, genes involved in multiple pathways that may be associated with T2DM phenotype were observed in GK rats. [20–22].

High-fat diet (HFD) is commonly used as experimental strategy to develop obesity and T2DM in rodents, simulating an environmental influence on the metabolism of these animals. HFD feeding was first described in C57BL/6 mice by Surwit et al [23], showing that HFD containing 58% of energy derived from fat leads to obesity, initial hyperinsulinaemia, impaired glucose homeostasis due to insulin resistance, and late insufficient insulin production due to β pancreatic cell failure [24]. These diets are enriched with saturated fat and promote weight gain by expansion of white adipose tissue (WAT), altering lipid homeostasis, adipocyte differentiation and survival. Consequently to these alterations, WAT expansion promotes an inflammatory state and leucocyte infiltration [25–27]. Chronic exposition to HFD induces liver damage [28], impaired glucose homeostasis, compensatory hyperinsulinemia to maintain normal glycemia (in the initial stage), late pancreatic β-cell failure to produce insulin due to cell exhaustion and consequent hyperglycemia, which are the main characteristics of T2DM [29–31].

Considering that the prevalence of T2DM is increasing globally and that this syndrome is a result from interactions of different factors, it is important to understand the altered mechanisms in T2DM and promote alternative options to minimize the consequences and progression of this disease. In this study we investigate the molecular mechanisms of insulin resistance induced by two different models: GK rats (genetic) and HFD inducing obesity (environmental—diet). We expect that the conclusion of this work help other researchers to choose their appropriate experimental model when aiming to study the causes and consequences of T2DM.

## Material and methods

### 1. Animals

Goto Kakizaki and Wistar rats were obtained from Charles River Laboratories International, Inc. (Wilmington, MA, USA) and maintained in our animal facility in the Department of Physiology and Biophysics of the Biomedical Sciences Institute, University of Sao Paulo. The rats were maintained at 23 ± 2°C under a cycle of 12 hours of light and 12 hours of darkness, being allowed free access to food and water. Male rats were fed with standard rodent chow (Nuvilab^®^, Curitiba, PR, Brazil) until 8 weeks of age. Then, the animals were randomly allocated into three groups: control and GK groups fed a control diet, and Wistar group fed a HFD (60%) (Table 1). Animals received the specific diets for eight weeks [32, 33]. Diets were obtained from Rhoster Company (Araçoiaba da Serra, SP, Brazil) and their composition was established based on Research Diets, Inc (New Brunswick, NJ, USA): D12450B (control) and D12492 (HFD). The Animal Ethical Committee of the Institute of Biomedical Sciences of the University of Sao Paulo (number 109/2013) approved all experimental procedures of this study. Body weight gain was evaluated weekly and food intake was measured three times a week.

**Table 1:** Composition of rodent diets.

Ingredients	Control Diet (10% Kcal of fat)	Hyperlipidic Diet (60% Kcal of fat)
Casein (80 mesh)	200 g (800 Kcal)	200 g (800 Kcal)
L-Cystein	3 g (12 Kcal)	3 g (12 Kcal)
Corn starch	315 g (1260 Kcal)	0 g (0 Kcal)
Maltodextrin 10	35 g (140 Kcal)	125 g (500 Kcal)
Sucrose	350 g (1400 Kcal)	68.8 g (275,2 Kcal)
Cellulose (BW200)	50 g (0 kcal)	50 g (0 kcal)
Soybean oil	25 g (225 Kcal)	25 g (225 Kcal)
Lard	20 g (180 Kcal)	245 g (2205 Kcal)
Mineral mix S10026	10 g (0 Kcal)	10 g (0 Kcal)
DiCalcium Phosphate	13 g (0 Kcal)	13 g (0 Kcal)
Calcium Carbonate	5.5 g (0 Kcal)	5.5 g (0 Kcal)
Potassium Citrate	16.5 g (0 Kcal)	16.5 g (0 Kcal)
Vitamin Mix V10001	10 g (40 Kcal)	10 g (40 Kcal)
Choline Bitartrate	2 g (0 Kcal)	2 g (0 Kcal)
Total	1055 g (4057 Kcal)	773.8 g (4057 Kcal)

### 2. Metabolic assays

#### 2.1. Glucose tolerance test (GTT) and insulin levels

After 12 h fasting, animals were injected (i.p.) with a 50% glucose solution, using a dose of 2 g/Kg (b.w.). Blood glucose concentration was determined using a blood glucose monitor (AccuCheck, Roche, SP, Brazil). Blood samples were obtained from a tail tip cut before (0 min) and at the following time points after glucose injection: 15, 30, 60, 90, and 120 min. Insulin level was measured by ELISA, according to the manufacture’s instructions (Merck Millipore, Darmstadt, Germany).

#### 2.2. Insulin tolerance test (ITT)

After 12 h fasting, animals were injected (i.p.) with insulin (Humulin R; Eli Lilly, Indianapolis, USA), using a dose of 0.5 IU/Kg (b.w.). Blood samples were obtained from a tail tip cut before (0 min) and at the following time points after insulin injection: 4, 8, 12, 15, 20 and 30 min. The constant rate for the insulin tolerance test (kITT) was calculated based on the linear regression of blood glucose concentrations obtained from 0 to 30 min of the ITT curve [34, 35].

#### 2.3. Measurement of plasma metabolites

Plasma FFA levels were determined using the enzymatic colorimetric assay (NEFA C) from Wako Chemicals GmbH, according to the manufacturer’s instruction. Plasma levels of triglycerides, total cholesterol, and HDL were determined using enzymatic colorimetric assays from BioClin (Minas Gerais, Brazil), according to the manufacturer’s instructions. LDL values were obtained by Friedewald equation [36]. HOMA-IR and HOMAb were calculated as described by Matthews et al. [37].

### 3. Lipid extraction and determination of plasma fatty acids composition by gas chromatography

Derivatization of plasma lipids was performed according to AOAC Official Methods 996.06 [38], with some modifications. Aliquots of plasma (150 μL) were added to a screw-cap test tube with 0.1 mL standard (5 mg/mL tritridecanoin C13:0 in chloroform) and 0.5 M NaOH in methanol. The tubes were placed in a water bath at 100°C, for 5 min. Methylation was performed by addition of 2 mL BF3-methanol (14%) and subsequent boiling at 100°C for 30 min. After cooling at room temperature, 1.5 mL isooctane and saturated NaCl were added to allow organic and watery phase separation. The organic phase with fatty acids was evaporated under N_2_ and 0.2 mL hexane was added to each sample. Samples were analyzed using gas chromatography on a GC 2012 plus (Shimadzu) equipped with a flame-ionization detector (FID), automatic injector AOC-20 and a Workstation Class GC10. Fatty acid separation was achieved using a fused-silica column SP-2560 (bis-cyano-propyl polysiloxane) [100 m (length) and 0.25 mm (diameter)]; Supelco, Bellefonte, USA). The column temperature was programed as follows: 140°C for 5 min; heating at 4°C/ min until 240°C; and 240°C for 30 min. The injector and detector were at 250°C, and helium was used as the carrier gas at a 1 mL/min flow rate. The split ratio was 1/50. Two microliters of derivatized lipid extract were injected and the fatty acid methyl ester peaks identified by comparison of retention times of fatty acid methyl ester standards and the chromatograms viewed in the Ce 1 h-05 methods [39].

### 4. Glutamyl oxaloacetic transaminase (GOT) and Glutamyl pyruvic transaminase (GPT) determination

GOT and GPT were measured in the plasma of 12 h fasted animals, using the colorimetric assay from LabTest (Minas Gerais, Brazil), according to the manufacturer’s instructions.

### 5. Insulin signaling in soleus skeletal muscle

After 4 h fasting, rats were anaesthetized with xilasine hydrochloride and ketamine solution by i.p. injection at 8 and 80 mg/kg b.w, respectively (Virbac do Brasil, São Paulo, Brazil). Soleus muscle was removed, carefully and rapidly isolated, and incubated as described previously by Crettaz et al. [40] and Challiss et al. [41] and routinely performed by our group [42, 43] Briefly, soleus muscles were preincubated at 35°C in Krebs-Ringer bicarbonate buffer, pH 7.4, and maintained for 30 min with 95% O_2_ and 5% CO_2_ containing 5.5 mM glucose, at 90 oscillations/min. After 30 min, muscles were transferred to vials containing Krebs buffer in the presence or absence of insulin (7 nM) and incubated for 20 min. After incubation, muscles were immediately homogenized in RIPA buffer (Thermo Scientific, Rockford, IL, USA) containing protease inhibitor cocktail (Roche, Basel, Switzerland), at 4°C, using a Polytron PT-MR 3100 (Kinematica AG, Luzern, Switzerland), operated at maximum speed, for 30 s. Tissue extracts were centrifuged at 10,000 x *g* at 4°C for 10 min, and the supernatants collected for western blotting analysis.

### 6. Insulin signaling in liver and adipose tissue

After 4 h fasting, rats were anaesthetized as described above. The abdominal cavity was accessed and a slice of the liver and portion of the retroperitoneal adipose tissue were removed and immediately homogenized in RIPA buffer (Thermo Scientific, Rockford, IL, USA), containing protease inhibitor cocktail (Roche, Basel, Switzerland) at 4°C, using a Polytron, as described above. After initial tissue removal (basal condition), the portal vein was accessed and 0.5 mL of insulin solution (prepared in 0.9% NaCl), containing a dose of 2 IU/Kg (b.w) was injected. After 60 s and 120 s, another slice of the liver and another portion of the retroperitoneal adipose tissue were removed (insulin-stimulated condition), respectively, and homogenized as described above. All tissue extracts were centrifuged at 10,000 x *g*, at 4°C, for 10 min, and the supernatants collected for western blotting analysis.

### 7. Western blot analysis

Protein content was determined in the supernatant of tissue extracts using BCA kit (Thermo Scientific, Rockford, IL, USA) and 4x Laemmli Sample Buffer [44] was added to the samples. Equal amounts of proteins (20 μg) were resolved in SDS-PAGE and transferred to nitrocellulose membranes. Membranes were blocked for 1h at room temperature with 5% skim milk and incubated with the specific primary antibodies overnight. Following incubation with secondary antibody conjugated to horseradish peroxidase. Bands were detected with the enhanced chemiluminescence system (Amersham Biosciences). Immunoblots were quantified using ImageJ^®^ software and Ponceau staining was used as an inner control [45, 46]. Phospho-AKT, phospho-GSK-3β, GSK-3β polyclonal antibodies were purchased from Cell Signaling (Danvers, MA, USA); AKT polyclonal antibody was purchased from Santa Cruz Biotechnology (Dallas, TX, USA); IL-1 β, TNF-α, IL-6 and IL-10 polyclonal antibodies were purchased from Abcam (Cambridge, MA, USA).

### 8. Histological analyzes of liver slices

#### 8.1. Morpho-quantitative evaluation

After collection and pre-fixation in 4% buffered paraformaldehyde, liver from the three experimental groups were dehydrated by a growing series of alcohols, diaphanized in xylol, embedded in paraffin and 4 μm liver sections were obtained and mounted onto silanized slides. Sections were stained by Hematoxylin & Eosin (HE) technique and used in the morphometric and quantitative evaluation of liver cells. The area (μm^2^) of the hepatocytes’ cellular and nuclear profiles were determined by measuring 50 cells and nuclei/animal, randomly selected, summing 250 cells/group. Cell density (cells/mm^2^) was determined as described by Mandarin-de-Lacerda [47], using 5 semi-serial sections/animal and 2 fields/section were analyzed, totalizing 10 photomicrographs/animal. Morpho-quantitative analyzes were performed using a computerized imaging device (Axio Vision 4.5 Zeiss ^®^) coupled to a 40x objective trinocular microscope (Zeiss Axiovert 40; Camera: Zeiss AxioCam ERc 5s).

#### 8.2. Hepatic fat accumulation

Liver was included in Tissue-Tek^®^ medium, frozen in liquid nitrogen and 10 μm sections were obtained and adhered onto silanized slides. Slides were fixed in 4% buffered paraformaldehyde, washed in distilled water and stained with Oil Red O and hematoxylin for lipid analysis. The qualitative evaluation of the slides was performed using photomicrographs captured by Zeiss Axiovert 40 microscope, with a 40x objective (Camera: Zeiss AxioCam ERc 5s).

#### 8.3. Hepatic glycogen content

Liver slides from frozen sectioning (10 μm) were stained with Periodic Acid-Schiff (PAS) and hematoxylin for the detection of hepatic glycogen. The qualitative evaluation of the slides was performed using photomicrographs captured by Zeiss Axiovert 40 microscope, with a 40x objective (Camera: Zeiss AxioCam ERc 5s).

### 9. Histological analyzes of inflammatory infiltrate in the retroperitoneal adipose tissue

Retroperitoneal adipose tissue was included in Tissue-Tek^®^ medium, frozen in liquid nitrogen and 5 μm sections were obtained and adhered onto silanized slides. Slides were stained with HE. Evaluation of leukocyte infiltrate was also performed by immunohistochemistry. Slides were blocked with 3% BSA and incubated overnight with mouse anti-rat CD11b/c mouse antibody (BD Pharmingen ^™^, 1:1000). Subsequently, the tissue was incubated with biotinylated anti-mouse IgG (Jackson ImmunoResearch, 1:200) secondary antibody for 2 hours, washed with 0.1M phosphate buffer and incubated with VectaStain ABC kit (Vector Laboratories, 1:100) for 2 hours. Detection of the antigen-antibody complex was performed through the chromogen 3,3'-diaminobenzidine (DAB) for 5 minutes at room temperature. Sections without the primary antibody (Cd11b/c) were used as negative control of the immunolabeling process. The qualitative evaluation of the slides was performed using photomicrographs captured by Zeiss Axiovert 40 microscope, with a 40x objective (Camera: Zeiss AxioCam ERc 5s).

### 10. Data analysis

Results are presented as mean ± S.E.M. Statistical significance was assessed by one-way or two-way ANOVA followed by the Bonferroni post-test. p ≤ 0.05 was considered statistically significant.

## Results

**Fig 1:**
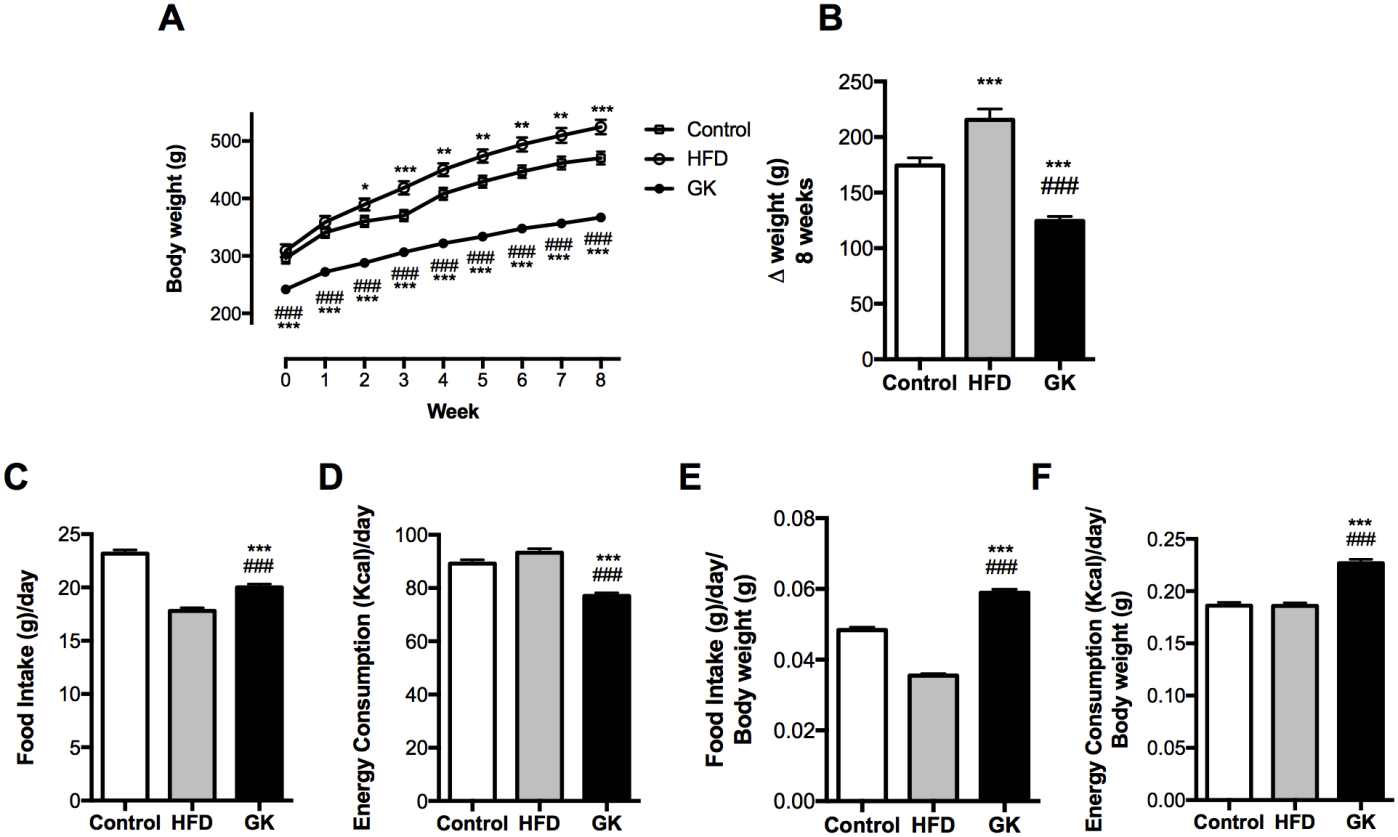
Body weight evaluation (A) and body weight gain (B) after 8 weeks of diet. Daily food intake (grams) (C); Daily energy intake (Kcal) (D); Food and energy consumption normalized by the animal’s body weight (E; F). Results are presented as mean ± S.E.M and n represents the number of animals used in each group. Studied groups: Control (n = 28), HFD (n = 22) and GK (n = 34). (*) p <0.05 *vs* control; (**) p <0.01 *vs* control; (***) p <0.001 *vs* control; (###) p <0.001 *vs* HFD.

HFD induced obesity in Wistar rats. GK rats were resistant to weight gain, showing, after 8 week treatment, 28% and 42% less gain when compared to the control group and HFD fed animals, respectively (Fig 1A and 1B). However, when energy consumption was measured, GK rats consumed more energy per day per Kg than the control and HFD groups, when food and energy intake were normalized by the body weight (Fig 1F). Even though HFD fed animals presented higher weight gain, HFD and control groups had equal amounts of energy consumption per day (Fig 1C–1F).

**Fig 2:**
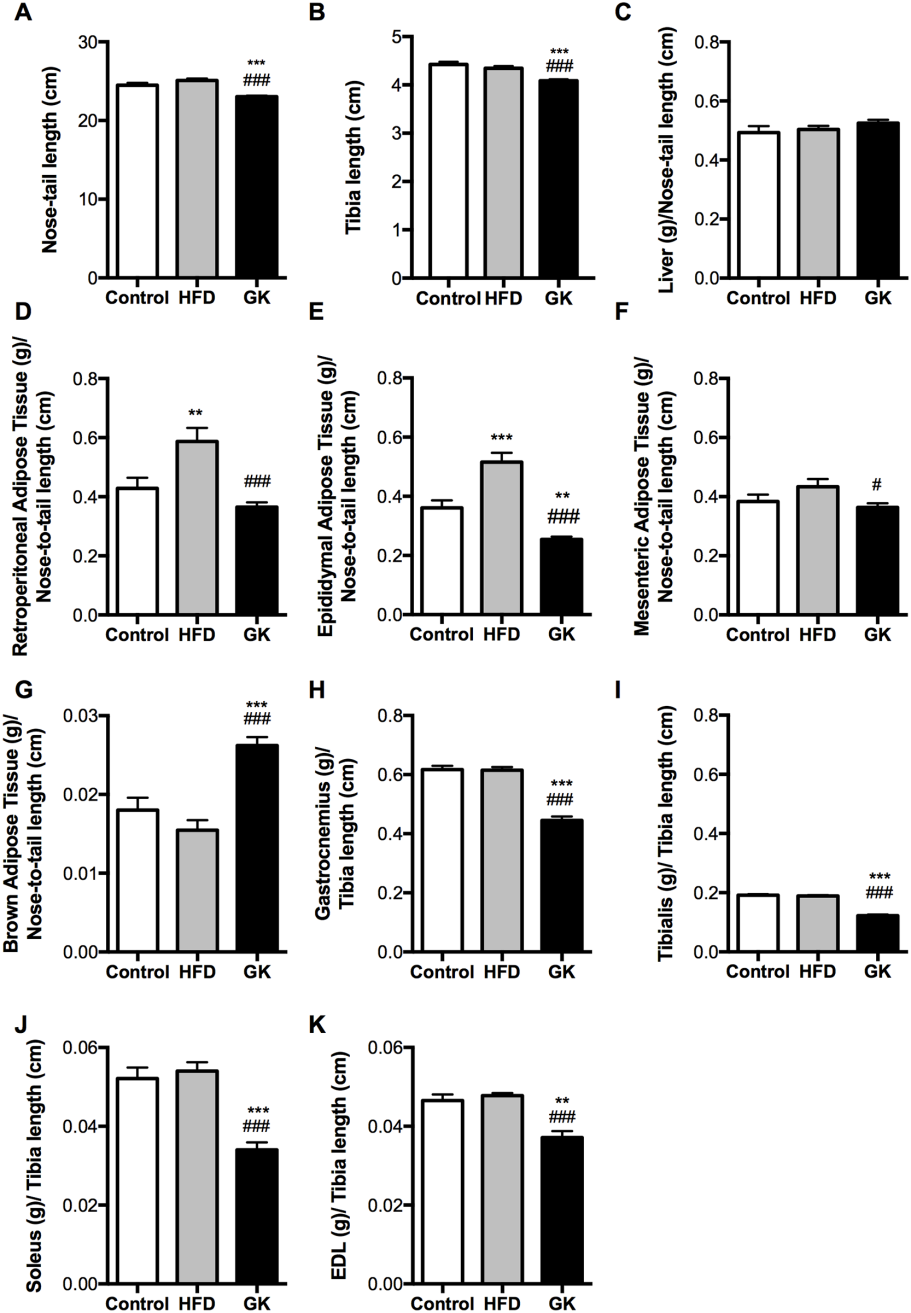
Nose-to-tail length (A); Tibia length (B); weight of Liver (C); Adipose tissues (D-G) and skeletal muscles (H-K). Adipose tissues and liver were normalized by the animal’s nose-to-tail length. Muscles were normalized by the animal’s tibia length. Results are presented as mean ± S.E.M and n represents the number of animals used in each group. Studied groups: Control (n = 9), HFD (n = 12) and GK (n = 21). (**) p <0.01 *vs* control; (***) p <0.001 *vs* control; (#) p <0.05 *vs* HFD; (###) p <0.001 *vs* HFD.

GK rats had decreased nose-to-tail length (Fig 2A), less adipose tissue and lower muscle humid weight when compared to control and HFD fed rats (Fig 2D–2K). Interestingly, brown adipose tissue was hypertrophied in GK rats (Fig 2G). Weight gain in the HFD group was mainly by the accumulation of fat in the retroperitoneal and epididymal adipose tissue (Fig 2D and 2E). No differences in liver weight were observed in the studied groups (Fig 2C).

**Fig 3:**
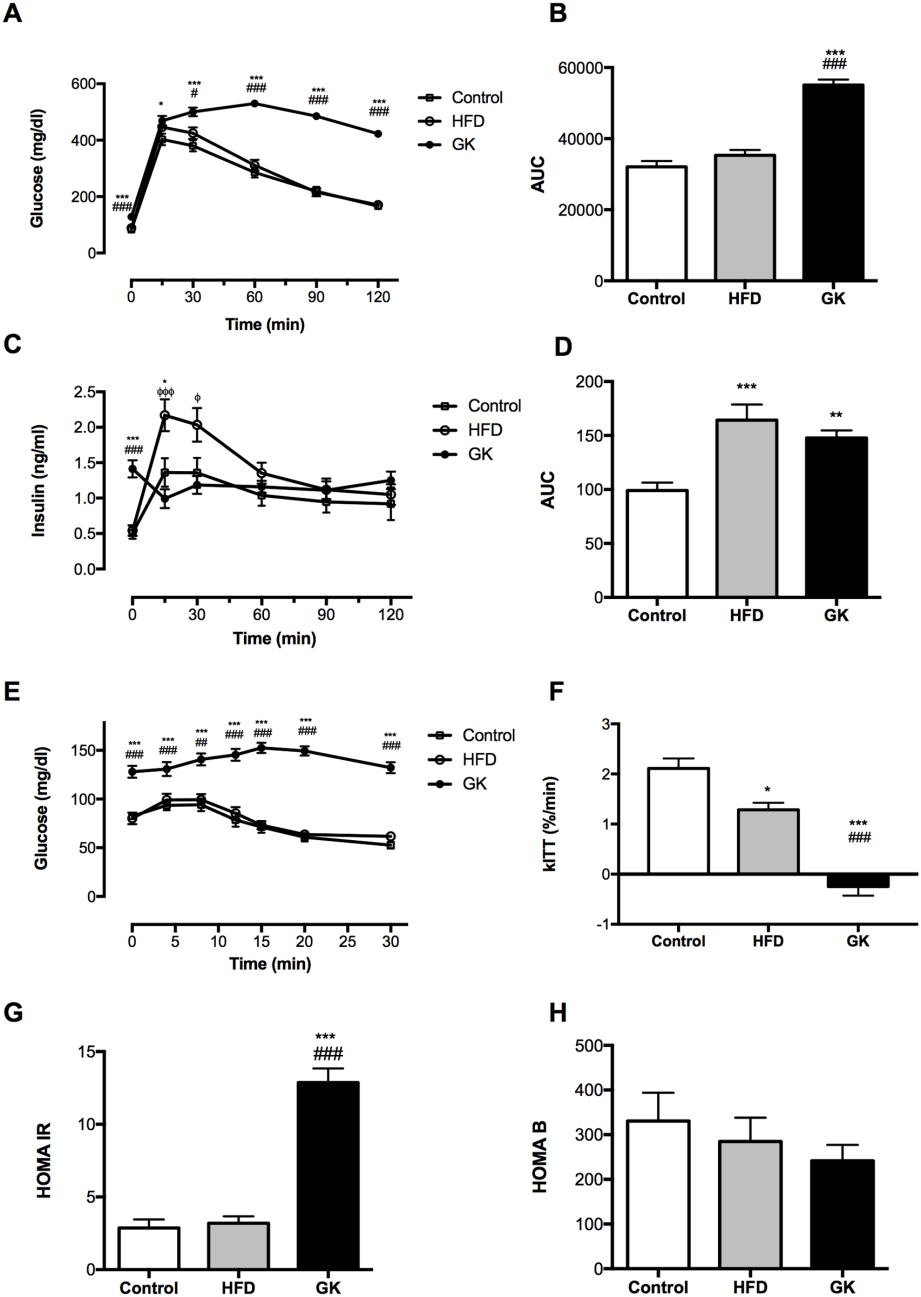
Glycemia measured during GTT (glucose 2 g/Kg (b.w) (i.p) after time 0 collected) (A); area under curve of glycemia measured during GTT (B); plasma insulin levels measured during GTT (C), area under curve of plasma insulin levels measured during GTT (D); glycemia measured during ITT (insulin 0.5 IU/Kg (b.w) (i.p) after time 0 collected) (E); rate constant for ITT—kITT (F); HOMA IR (G) and HOMA B (H) indexes. Results are presented as mean ± S.E.M and n represents the number of animals used in each group. Studied groups: Control (n = 24), HFD (n = 17) and GK (n = 31). (*) p <0.05 *vs* control; (**) p <0.01 *vs* control; (***) p <0.001 *vs* control; (#) p <0.05 *vs* HFD; (###) p <0.001 *vs* HFD; (ϕ) p<0.05 *vs* GK; (ϕϕϕ) p<0.001 *vs* GK.

When animals were glucose challenged (2 g/kg b.w), GK rats reached a glycemic peak after 60 min and maintained high plasma glucose concentration until the end of the experiment (120 min) (Fig 3A and 3B). Control and HFD fed rats reached a glycemic peak at 15 min and glucose plasma concentration decreased gradually until 120 min (Fig 3A and 3B). Before the glucose challenge, GK rats presented fasting hyperglycemia and hyperinsulinemia when compared to the other groups (Fig 3A and 3C). Also, these animals failed to secrete/produce insulin after glucose stimulus (Fig 3B). On the other hand, HFD fed rats secreted a great amount of insulin after glucose challenge (Fig 3C and 3D). In the insulin tolerance test (ITT), GK rats didn’t respond to insulin, maintaining high glucose plasma concentration after insulin stimulus (Fig 3E). HFD fed animals presented decreased insulin sensitivity (kITT) when compared to the control group and GK rats showed insulin resistance of greater magnitude (Fig 3F). HOMA-IR and HOMA-B is clearly altered in the GK group (Fig 3G and 3H).

**Fig 4:**
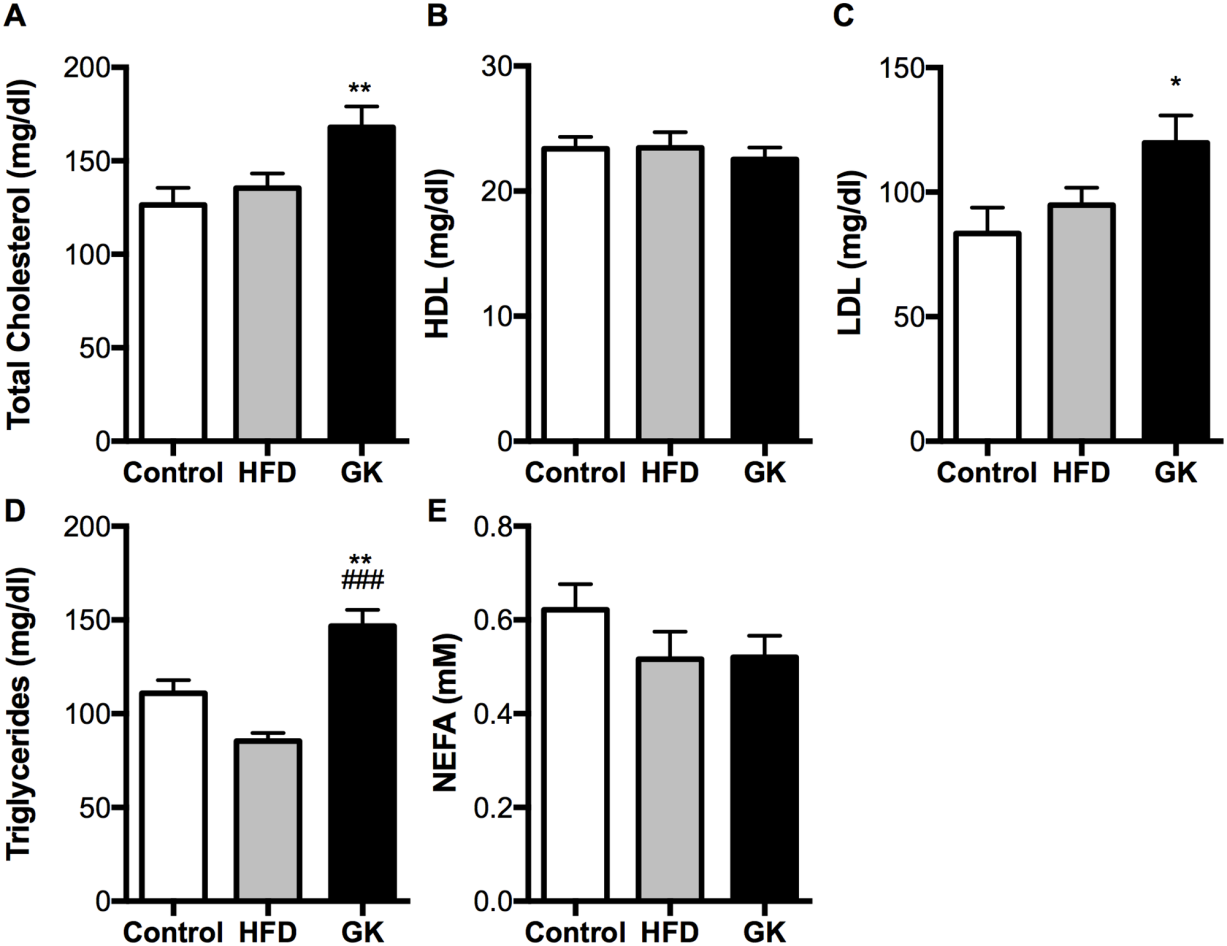
Total cholesterol (A); HDL (B); LDL (C); triglycerides (D); NEFA (E). Six hours after instillation of saline or LPS, animals were anesthetized and the blood was collected through the abdominal aorta. The dosages were carried out in the plasma by enzymatic-colorimetric method. Results are presented as mean ± S.E.M and n represents the number of animals used in each group. Studied groups: Control (n = 24); HFD (n = 20) and GK (n = 27). (*) p <0.05 *vs* control; (**) p <0.01 *vs* control; (###) p <0.001 *vs* HFD.

Fasting cholesterol, LDL and triglycerides were increased in GK plasma and HFD didn’t alter these parameters (Fig 4). Gas chromatography (GC) showed higher contents of fat in plasma from GK rats (S1 Fig) and different fat profiles between the groups: HFD fed rats presented more saturated and less monounsaturated fats when compared to the control group (S1 Fig) and GK animals had higher contents of monounsaturated and lower contents of polyunsaturated fats when compared to the control group (S1 Fig). No difference was observed in plasma leptin and adiponectin among the studied groups (S2 Fig). GOT and GPT levels were higher in the plasma of GK rats (S3 Fig).

**Fig 5:**
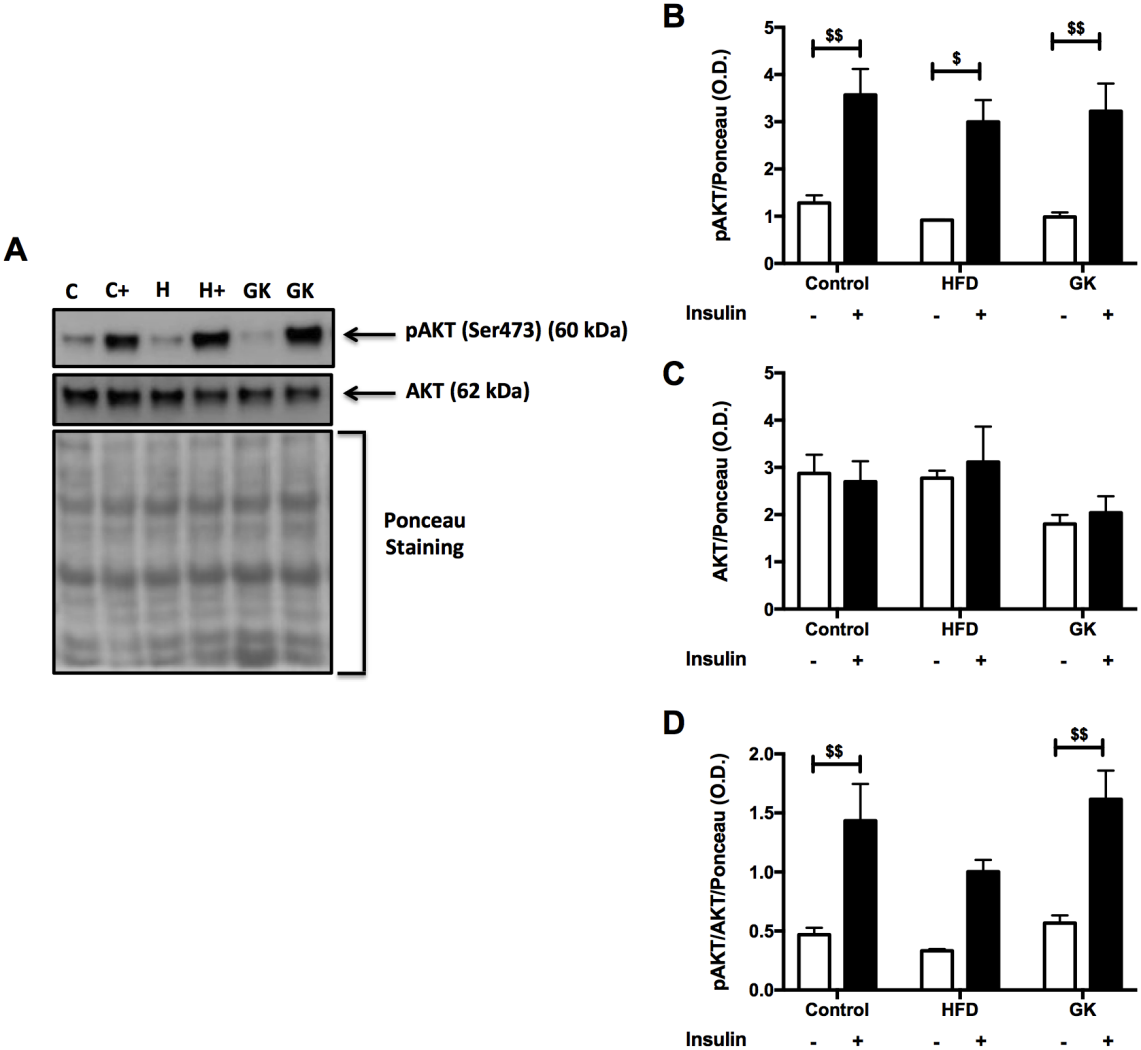
pAKT and AKT quantification in soleus muscle by WB (A-D). Positive stimulus: insulin (7 nM). Graphs present mean O.D. ± S.E.M of the bands and n represents the number of animals used in each group. Studied groups: Control (n = 5); HFD (n = 3) and GK (n = 5). ($) p <0.05; ($ $) p <0.01 as indicated in the graphs.

**Fig 6:**
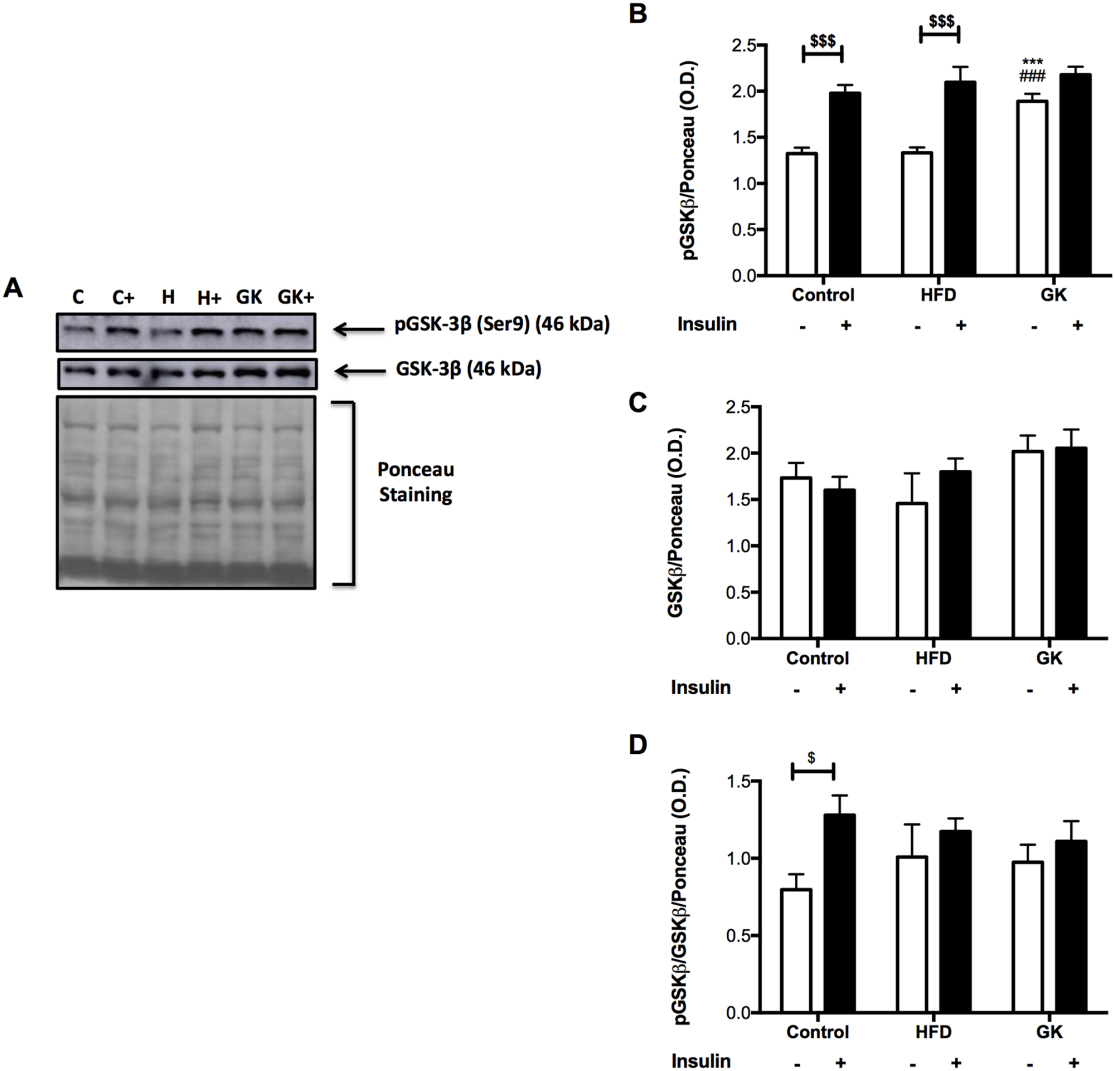
pGSK-3β and GSK-3β quantification in soleus muscle by WB (A-D). Positive stimulus: insulin (7 nM). Graphs present mean O.D. ± S.E.M of the bands and n represents the number of animals used in each group. Studied groups: Control (n = 5); HFD (n = 3) and GK (n = 5). (***) p <0.001 *vs* control; (###) p <0.001 *vs* HFD; ($) p <0.05; ($ $ $) p <0.001 as indicated in the graphs.

Soleus muscle, retroperitoneal adipose tissue and liver were analyzed before and after insulin stimulus in order to verify the integrity of insulin response. In soleus muscle, after *in vitro* insulin stimulus, the content of pAKT augmented in all groups (Fig 5A). Total AKT content was similar in all studied groups and pAKT/AKT ratio was lower in the HFD group (Fig 5B and 5D). The pGSK-3β content didn’t increase after insulin stimulus in GK rats. pGSK-3β content, before insulin stimulus, in this group was already higher when compared to the other groups (Fig 6A). pGSK-3β/GSK-3β ratio was altered in the HFD and GK groups, not showing an increase after insulin stimulus as observed in the control group (Fig 6D).

**Fig 7:**
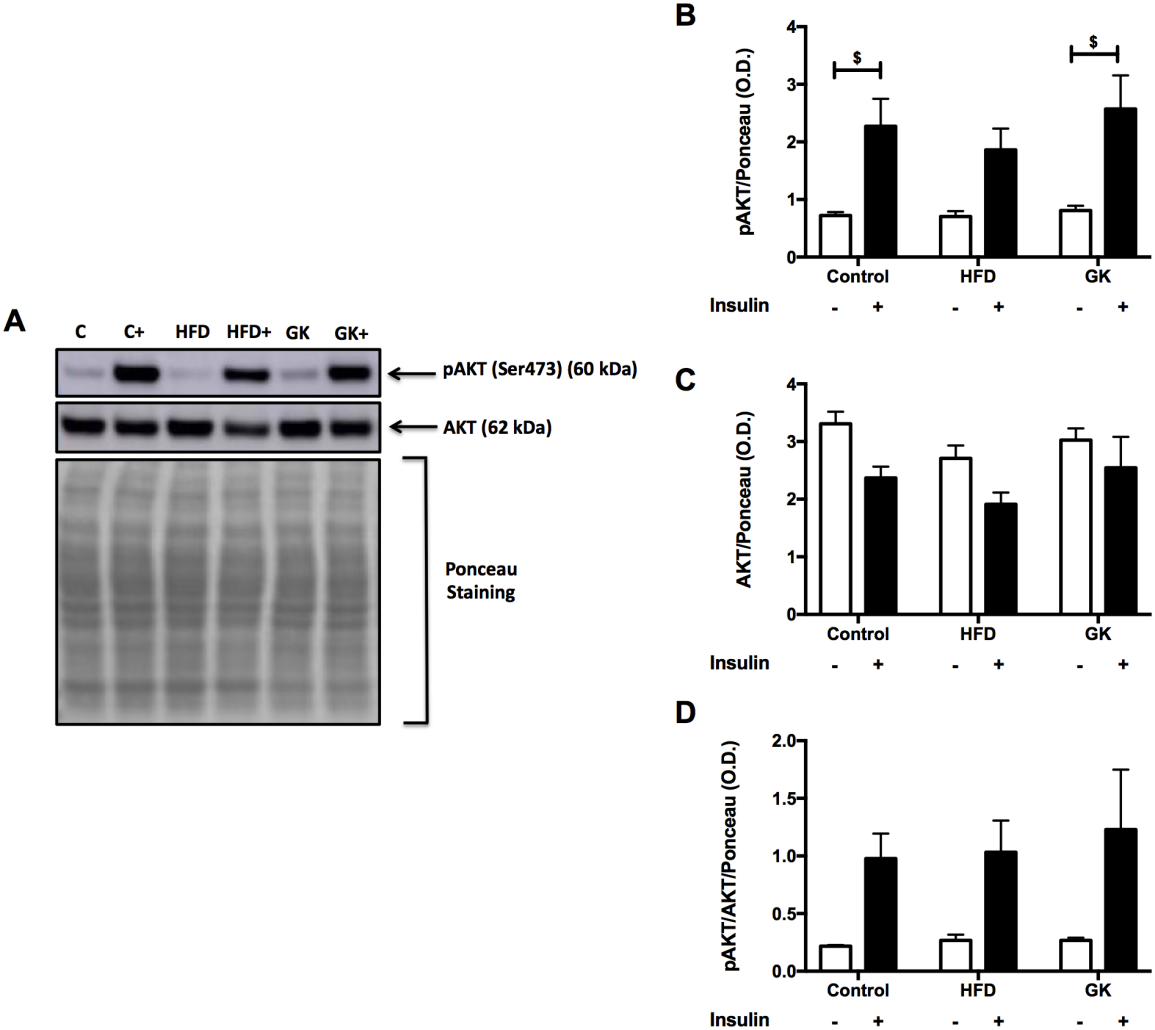
pAKT and AKT quantification in liver by WB (A-D). *In vivo stimulus*: insulin 2IU/Kg (b.w) (i.v.). Graphs present mean O.D. ± S.E.M of the bands and n represents the number of animals used in each group. Studied groups: Control (n = 3); HFD (n = 3) and GK (n = 3). ($) p <0.05 as indicated in the graphs.

**Fig 8:**
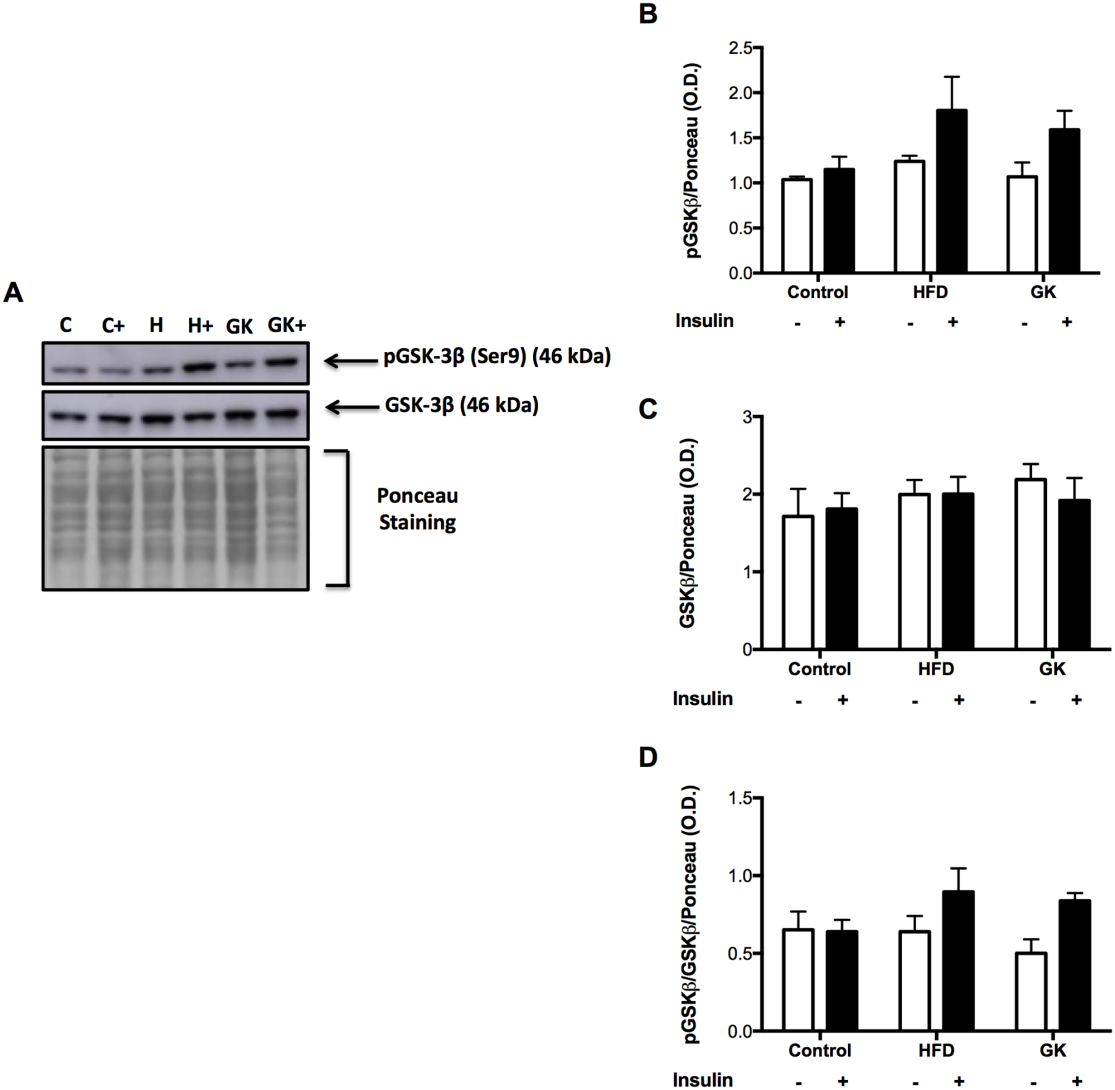
pGSK-3β and GSK-3β quantification in liver by WB (A-D). *In vivo stimulus*: insulin 2IU/Kg (b.w) (i.v.). Graphs present mean O.D. ± S.E.M of the bands and n represents the number of animals used in each group. Studied groups: Control (n = 3); HFD (n = 3) and GK (n = 3).

In the liver, HFD group didn’t show difference in pAKT content after *in vivo* insulin challenge (Fig 7A and 7C). Liver of the GK group responded equally to the control group (Fig 7A and 7C). pGSK-3β content and pGSK-3β/ GSK-3β ratio was statistically similar among all groups (Fig 8A–8D).

**Fig 9:**
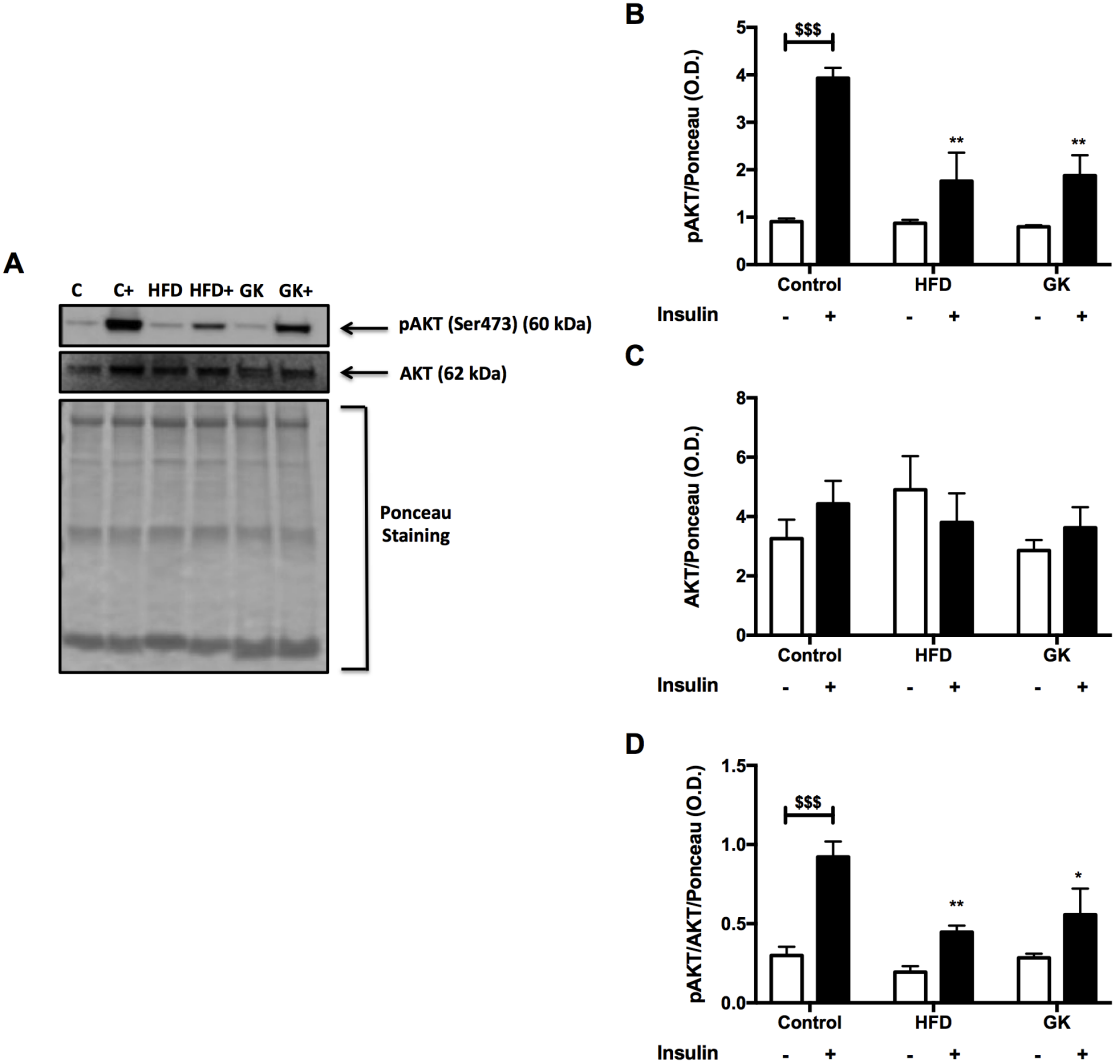
pAKT and AKT quantification in retroperitoneal adipose tissue by WB (A-D). *In vivo stimulus*: insulin 2IU/Kg (b.w) (i.v.). Graphs present mean O.D. ± S.E.M of the bands and n represents the number of animals used in each group. Studied groups: Control (n = 3); HFD (n = 3) and GK (n = 3). (*) p<0.05 *vs* control (+); (**) p<0.01 *vs* control (+); ($ $ $) p<0.001 as indicated in the graphs.

**Fig 10:**
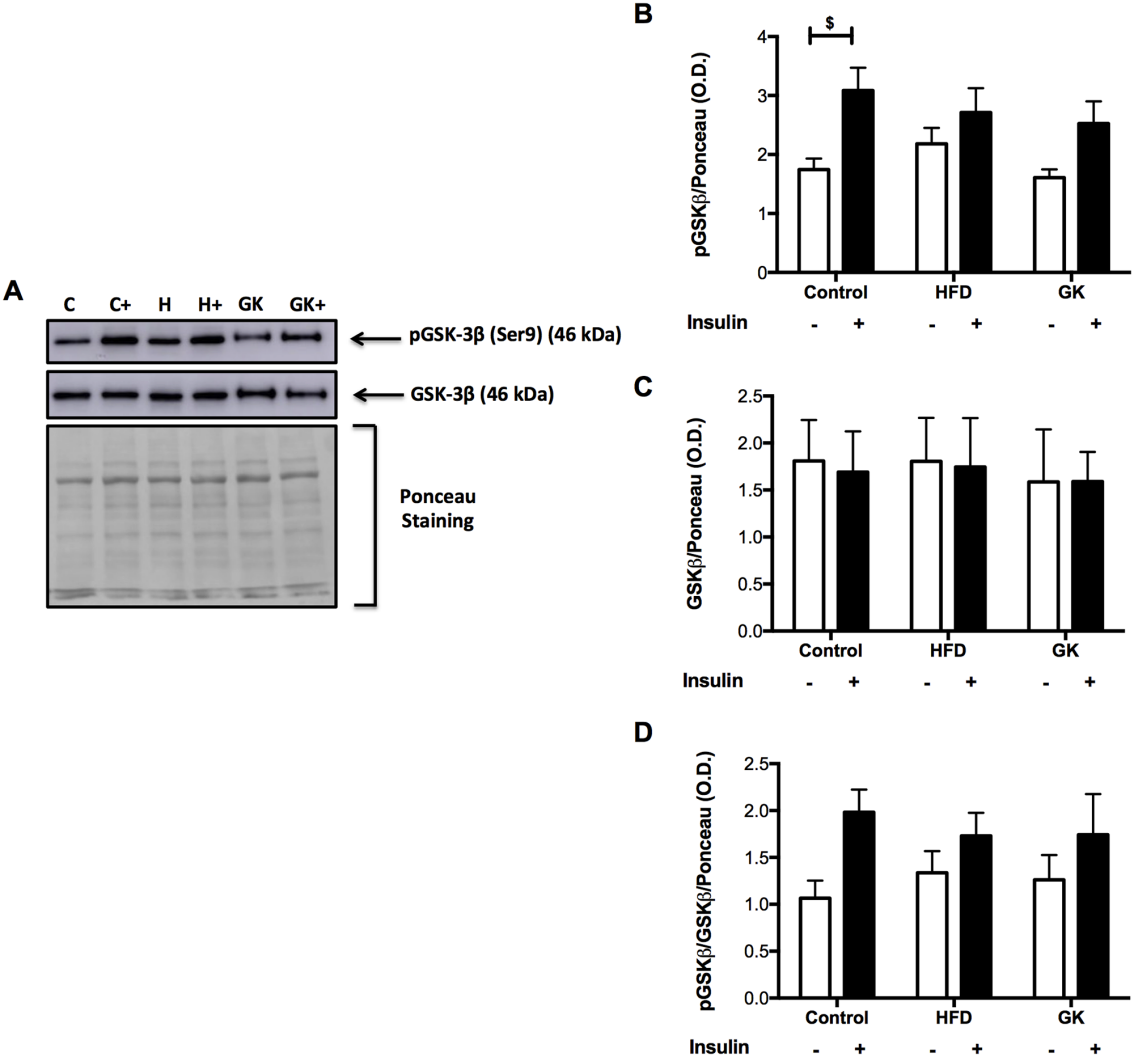
pGSK-3β and GSK-3β quantification in retroperitoneal adipose tissue by WB (A-D). *In vivo stimulus*: insulin 2IU/Kg (b.w) (i.v.). Graphs present mean O.D. ± S.E.M of the bands and n represents the number of animals used in each group. Studied groups: Control (n = 4); HFD (n = 4) and GK (n = 4). ($) p <0.05 as indicated in the graphs.

Retroperitoneal adipose tissue from GK and HFD fed rats presented lower pAKT content and lower pAKT/AKT ratio, after *in vivo* insulin challenge, when compared to the control group (Fig 9A and 9D). Accordingly to this result, pGSK-3β content did not increase after insulin stimulus in WAT of GK and HFD groups. pGSK-3β/ GSK-3β ratio were similar among all groups (Fig 10A–10D).

**Fig 11:**
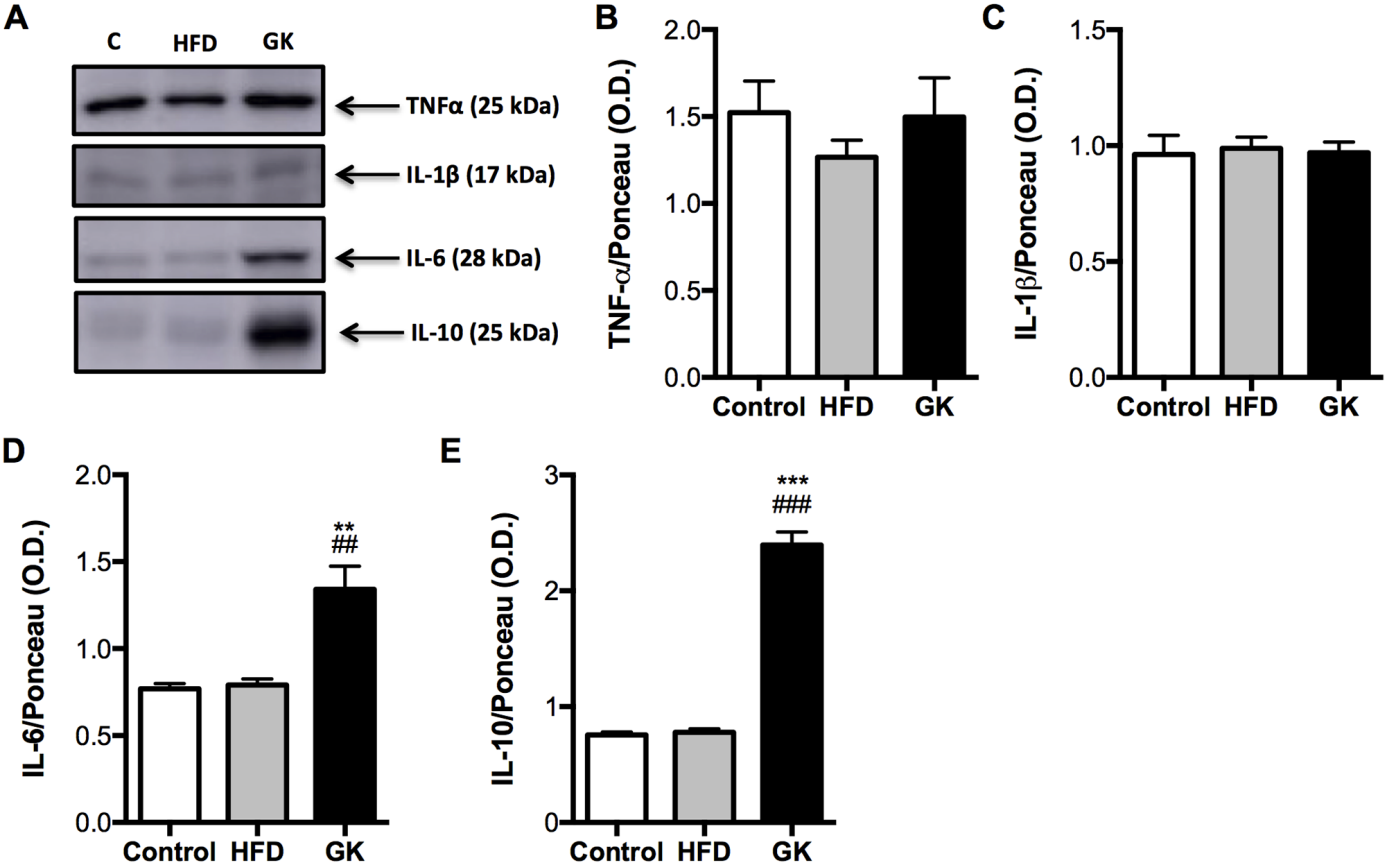
Content of IL-10, IL-6, TNF-α and IL-1β in liver. Graphs present mean O.D. ± S.E.M of the bands and n represents the number of animals used in each group. Studied groups: Control (n = 4); HFD (n = 4) and GK (n = 4). (**) p <0.01 *vs* control; (***) p <0.001 *vs* control; (##) p <0.01 *vs* HFD; (###) p <0.001 *vs* HFD. Ponceau staining used in the normalization of the blots are in S11 Fig.

**Fig 12:**
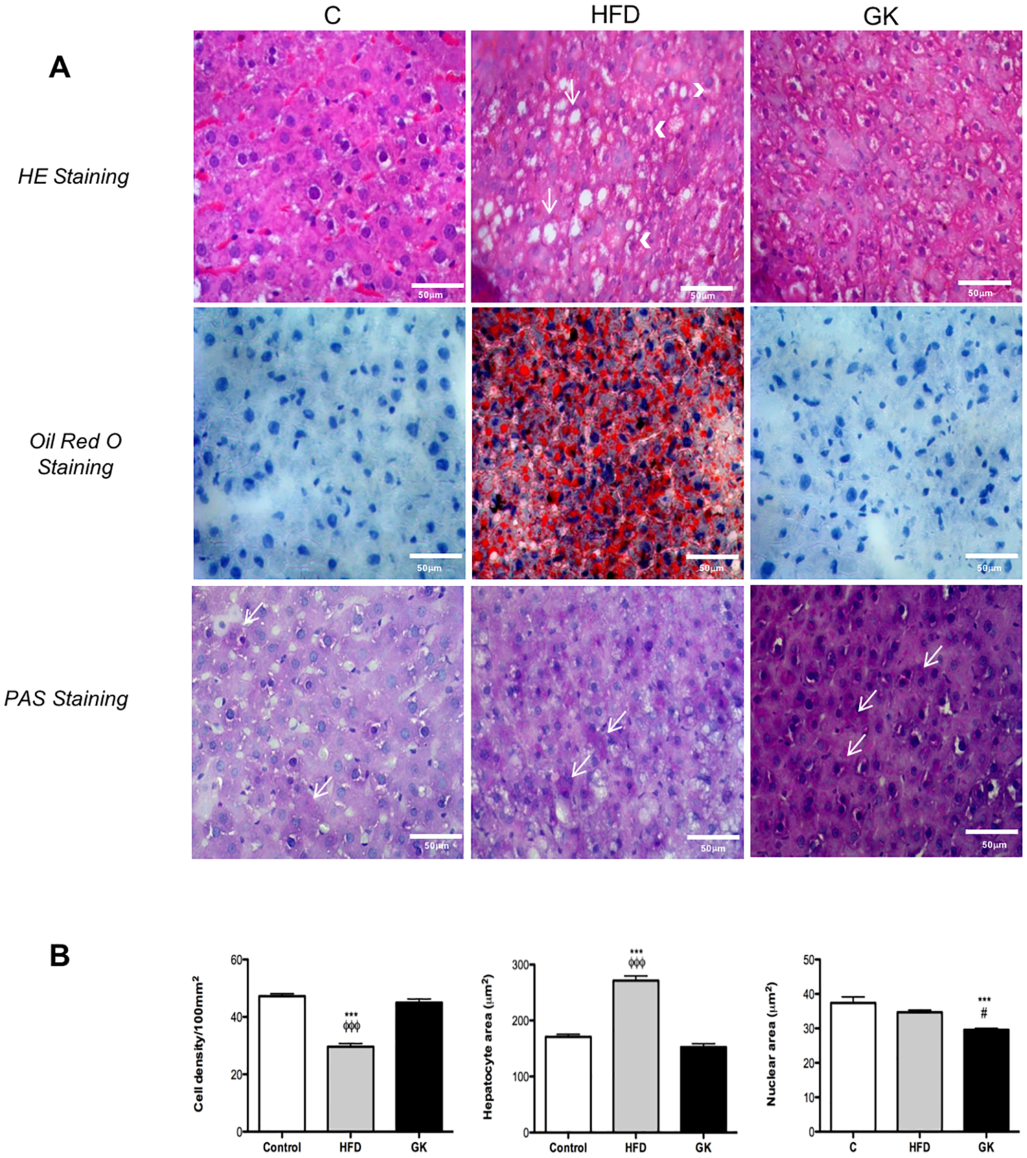
Histological analysis of the liver (A). HE and Oil Red staining revealed micro and macro steatosis (arrows) in HFD fed rats. PAS revealed high concentration of glycogen in liver from GK rats. Morpho-quantitative evaluations (B) considered cell density, hepatocyte and nuclear areas. Results are presented as mean ± S.E.M and n represents the number of animals used in each group. Studied groups: Control (n = 6); HFD (n = 6) and GK (n = 6). (***) p <0.001 *vs* control; (#) p <0.05 *vs* HFD; (ϕϕϕ) indicates p <0.001 *vs* GK. Calibration bar: 50μm.

In order to verify if these alterations in insulin response was due to an inflammatory process, cytokines were measured in the same tissues. No differences in TNF-α, IL1-β, IL-6 and IL-10 content were observed in soleus muscle among the studied groups (S4 Fig). In the liver, no difference in cytokines content was observed in the HFD group when compared to the control group (Fig 11A–11E) and, in the GK group, IL-6 and IL-10 content were increased when compared to the other groups (Fig 11D and 11E). Morphologically, liver from HFD fed rats had lower density of cells and higher hepatocytes area while liver from GK rats presented lower nuclear area (Fig 12A and 12B). When stained with oil red, hepatocytes from HFD group showed great accumulation of fat and hepatocytes from GK rats didn’t show any difference from the control group (Fig 12A).

**Fig 13:**
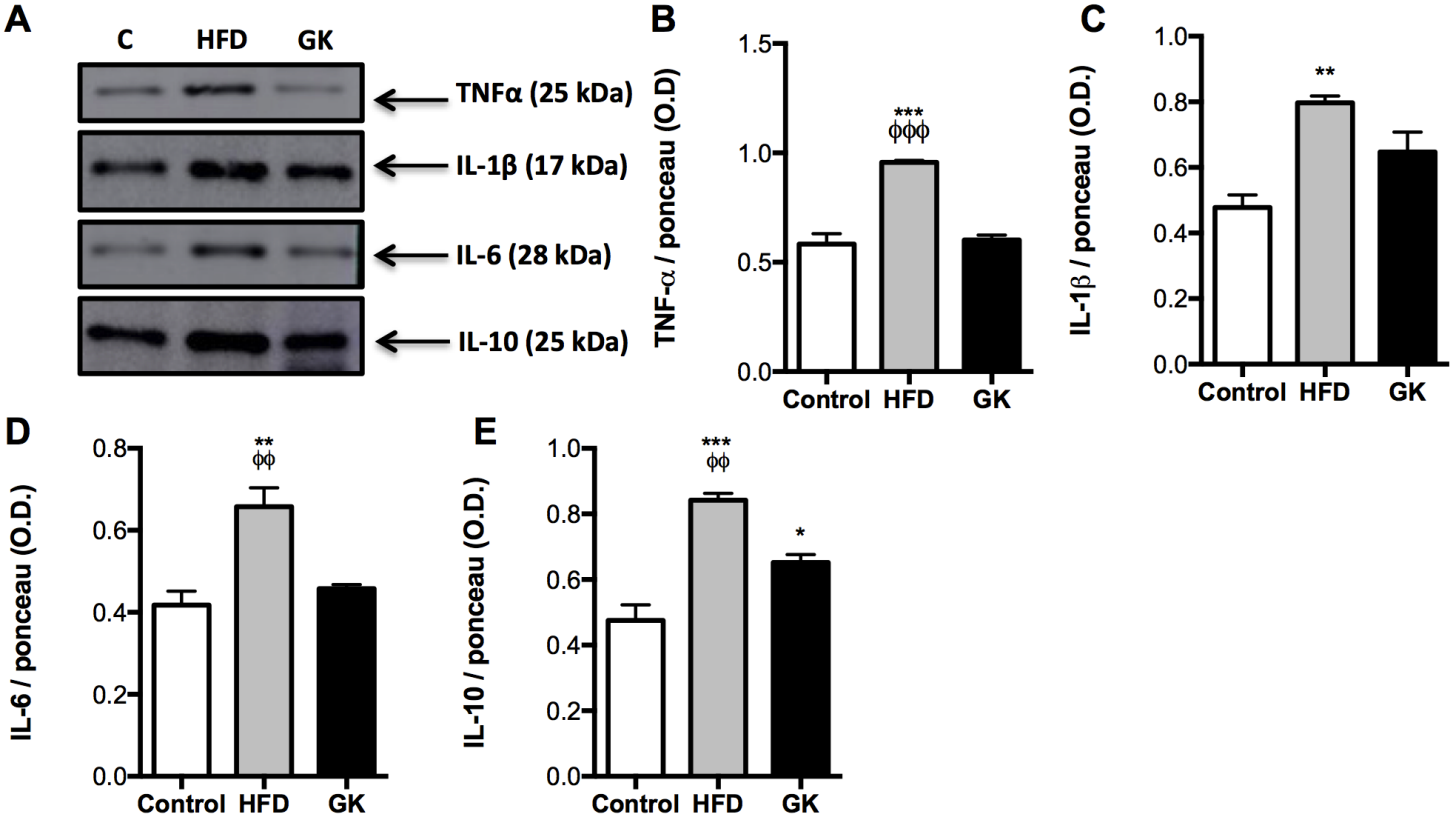
Contents of IL-10, IL-6, TNF-α and IL-1β in the retroperitoneal adipose tissue. Graphs present mean O.D. ± S.E.M of the bands and n represents the number of animals used in each group. Studied groups: Control (n = 4); HFD (n = 4) and GK (n = 4). (*) p <0.05 *vs* control; (**) p <0.01 *vs* control; (***) p <0.001 *vs* control; (ϕϕ) indicates p <0.01 *vs* GK; (ϕϕϕ) indicates p <0.001 *vs* GK. Ponceau staining used in the normalization of the blots are in S12 Fig.

**Fig 14:**
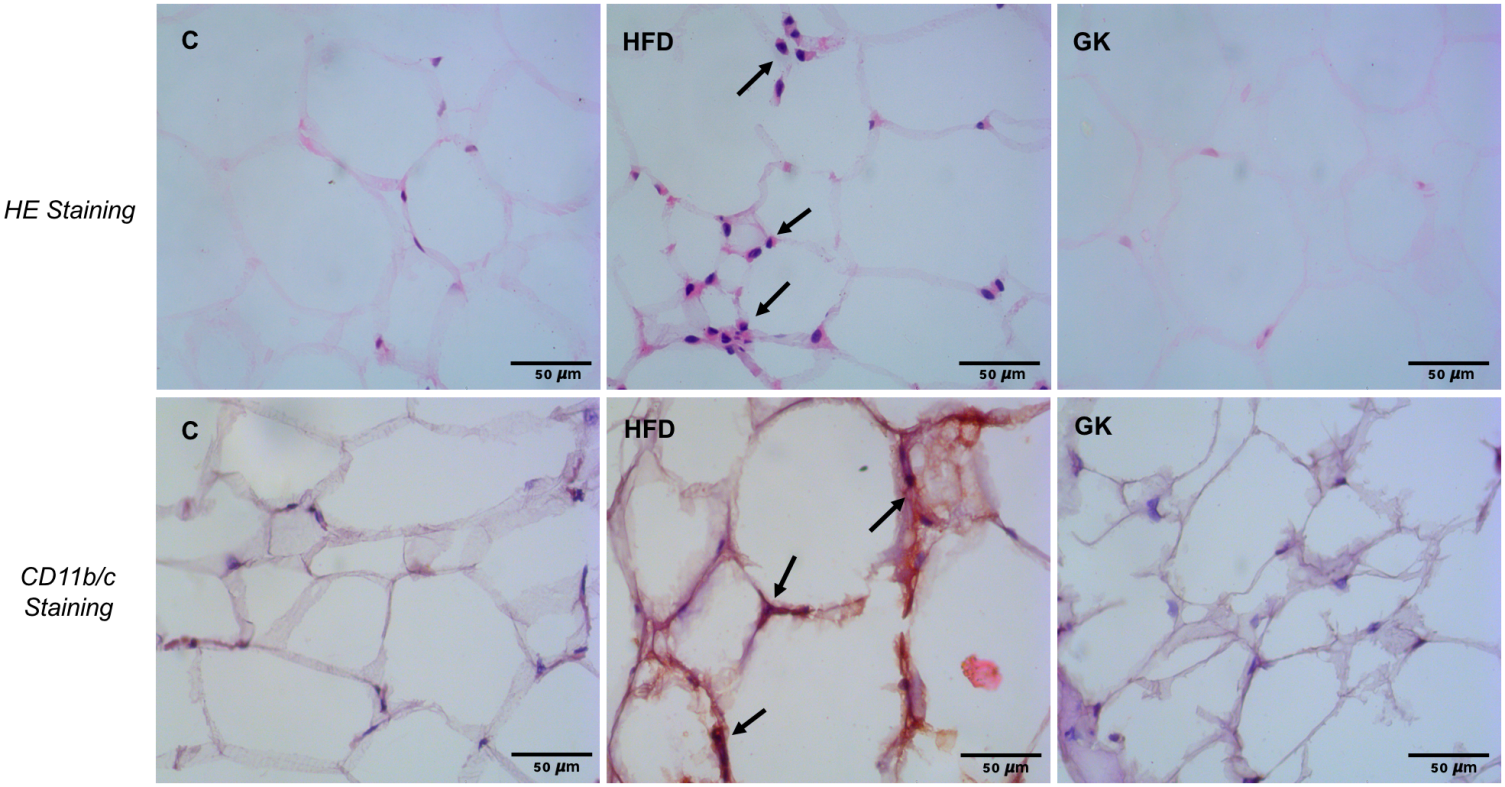
Histological analysis of retroperitoneal adipose tissue. Both HE staining (A) and the immunohistochemistry to CD11b/c revealed mononuclear cell infiltrate (arrows) around the adipocytes in the HFD group. Results are presented as representative images and n represents the number of animals used in each group. Studied groups: Control (n = 6); HFD (n = 6) and GK (n = 6). Calibration bar: 50μm.

HFD fed animals presented inflamed retroperitoneal adipose tissue, showing increase in TNF-α, IL-1β, IL-6 and IL-10 content when compared to the other groups (Fig 13A–13E). IL-10 was increased in retroperitoneal adipose tissue from GK rats (Fig 13E). Greater inflammatory infiltrate was observed in the adipose tissue from HFD fed animals and no differences in the amount of immune infiltrate was observed in GK rats (Fig 14).

## Discussion

As obesity grows throughout the world, T2DM becomes one of the leading causes of death, reaching different societies and cultures [1]. As well described by the scientific literature, obesity is strongly associated to the development of metabolic complications leading to T2DM in the occident [48]. However, not all type 2 diabetics individuals are obese. In the orient, especially Japan, India and China, half of the population that develops T2DM is considered lean (BMI < 25) [49, 50]. Even though obesity is a major cause of chronic inflammation associated to T2DM, patients that present a chronic inflammatory condition, such as periodontal disease, obstructive pulmonary disease, arthritis, myotonic dystrophy or chronic hepatitis C infection may also develop T2DM [51]. The difference in the genesis of this syndrome occurs because it depends not only on genetic background, but also on behavioral and environmental influence on the life of a certain population. The increase of T2DM prevalence is attributed to a sedentary lifestyle and unhealthy diet consumption, associated to genetic predisposition [52]. Both behavioral and environmental factors may be controlled to promote the reduction of obesity and associated comorbidities. Genetics is the only cause that still cannot be permanently changed.

Two models of rats, aiming two different approaches, were used to investigate their reliability as T2DM experimental models: the genetic (GK rats) and the environmental (obese HFD-induced wistar rats) influences in the development of TD2M. As a T2DM genetic susceptible model, GK rats were used. Various works have described the genetic differences of this strain that lead them to the diabetic phenotype [20–22]. These animals develop T2DM spontaneously without the interference of obesity and have been widely used to understand the mechanisms of pancreatic beta cell failure in producing insulin and its short/long term complications [9–14]. GK rats are also insulin resistant, but the pathways responsible for this resistance are not completely elucidated yet. On the other hand, in order to investigate the influence of external factors without the internal imprint in the possible development of T2DM, we used an obesity model induced by HFD. Diet composition, such as increase in fat or/and sugar, is strongly associated to the loss of insulin sensitivity in animal models, including Wistar rats [53]. Therefore, herein, we evaluated the glucose homeostasis, the liver and also two peripheral tissues, soleus muscle and retroperitoneal white adipose tissue, in response to the intrinsic (genetic) and extrinsic (HFD) influences.

Soleus muscles of GK responded equally to insulin stimulus to phosphorylate AKT in the serine site. Skeletal muscle is the most important peripheral tissue that controls glycaemia in mammal’s organism. Even though our results did not indicate any failure in AKT serine site phosphorylation in soleus of GK rats after insulin stimulus, we observed higher content of pGSK-3β before insulin stimulus. The hormone did not increase GSK-3β phosphorylation in soleus of GK rats as observed in the other groups. In normal insulin signaling, pAKT phosphorylates GSK-3β to transmit insulin signal through the cell. In soleus from GK rats, this signal seems to be deregulated, since pAKT is not able to phosphorylate GSK-3β because this protein is already phosphorylated at a high level. GSK-3β may be phosphorylated by different proteins besides pAKT, such as AMPK, PKA, PKC, p70 S6 kinase, and other kinases [54]. Further studies are necessary to address this point and to determine the involvement of GSK-3β signaling deregulation in the insulin resistance development in GK rats. Other mechanisms may be involved in the insulin resistance in skeletal muscle of these animals. For example, defects in muscle microvasculature in GK rats can contribute to an impairment of muscle cells metabolism and function [55]. Moreover, previous study by Dadke et al. reported that skeletal muscle of GK rats has augmented activity of tyrosine phosphatase 1B (PTP1B) before and after insulin stimulus. PTP1B is known to cause dephosphorylation of tyrosine sites and consequently acts as negative regulator of insulin signaling by inactivating insulin-signaling receptors [56]. HFD did not induce any alteration in soleus muscle insulin signaling, showing that 8 weeks of the diet administration was insufficient to cause impairment in glucose homeostasis in this tissue.

Adipose tissue is also an important site that controls glucose homeostasis in mammals. Although HFD did not induce hyperglycemia, as also observed by others [57, 58], it triggered insulin resistance in Wistar rats. HFD induced obesity with expansion of WAT depots. This WAT expansion was accompanied by an increase in leukocytes infiltration and inflammation in this tissue. Accordingly to our results, other groups also reported increase in the expression of inflammatory genes in WAT after HFD feeding [25, 26]. During obesity, leukocyte infiltration in WAT, composed mainly by macrophages, enhances TNF-α and IL-6 production, to initiate and maintain the inflammatory process [59, 60]. Although Wernstedt Asterholm et al. [61] show that adipose tissue inflammation is an adaptive response that is essential for storage of excess nutrients and that it contributes to WAT expansion and remodeling during HFD feeding, constant inflammatory stimulus may lead to insulin signaling impairment and consequently insulin resistance [59, 62].

Cytokines promote serine phosphorylation of insulin receptor substrate-1 (IRS-1) and impair insulin signaling by impeding the PI3K pathway [63, 64]. Inflammation was probably the main cause of insulin resistance in WAT in obesity model induced by HFD, since a concomitant increase in cytokines content and decrease in AKT phosphorylation in WAT was also observed in the present study. Even though HFD fed animals did not present an established insulin resistance in soleus skeletal muscle, WAT insulin resistance was punctually identified. HFD fed rats did not present glucose intolerance, however, these animals showed a great increase in insulin secretion after glucose stimulus, indicating that these animals present lower insulin sensitivity. This data was confirmed when kITT was calculated and corroborates with the WAT alterations.

Retroperitoneal white adipose tissue (rWAT) of GK rats had lower phosphorylation of AKT after insulin stimulus, indicating, again, the peripheral resistance to insulin in this animal model. Contrary to the HFD fed rats, GK rats have lower fat accumulation in the white adipose tissue depots. This result may be explained due to impairment in differentiation of pre-adipocyte into mature adipocyte, leading to a defect in triglycerides storage and increase in free fatty acids release to the plasma [65–67]. The impaired adipocyte differentiation observed in GK rats may be a consequence of chronic inflammation observed in the WAT of these animals [68]. Our results confirmed an increase in IL-10 content in the rWAT of GK rats, showing the establishment of a long-term inflammatory process at this site. Other mechanisms can contribute to insulin resistance in rWAT and are being better investigated by other researchers in our group. Furthermore, our results evidenced the impediment that GK rats present to decrease plasma glucose concentration and augment plasma insulin levels after a glucose stimulus, indicating deficiencies in the production and secretion of insulin by pancreatic β cells. Impairment in insulin production and secretion by pancreatic β cells from GK rats are also observed and well established by other groups in the literature [9–14]. Nevertheless, it is important to consider that elevated basal hepatic glucose production as a consequence of decreased insulin suppressive effect on hepatocytes, contribute to high glucose levels in plasma from GK rats [16, 69, 70].

Hyperglycemia *per se* is capable to promote inflammation in GK liver by altering the expression of genes that control pro and anti-inflammatory cytokines [70]. Our results confirmed an inflammatory state in the liver of GK rats by showing augmented IL-6 and IL-10 content at this site. Also, higher content of glycogen and no fat accumulation in liver from GK rats was observed in our histological analysis. Accordingly to our findings, Almon et al demonstrated that chronic hyperglycemia promotes deposition of glycogen and impedes excessive fat accumulation in the liver of GK animals [71]. Although Karpe et al [72] and our present work described that under fasted condition, plasma FFA levels were not different between the control and the HFD groups, Liu et al. reported that in the fed state, HFD promotes elevated circulating FFA levels as a result of increased dietary intake of lipids and impaired ability of postprandial insulin to effectively inhibit lipolysis [73]. Furthermore, it is also important to consider plasma FFA composition. Our results demonstrated that HFD animals present high percentage of saturated fatty acids (SFA) and low of monounsaturated fatty acids (MUFA) in plasma. This difference may be a consequence of a decrease in enzyme SCD1 activity, predominantly expressed in the liver, which converts SFA derived from dietary FA or from *de novo* lipogenesis into MUFA, as described by Paton & Ntambi [74]. Elevated SFA and decreased SCD1 activity in liver results in hepatocellular apoptosis, steatohepatitis and fibrosis [75]. Low TG levels observed in HFD fed animals may be explained by the higher accumulation of fat in liver, as previously demonstrated by Pan et al. [76] and Liu et al. [72].

**Fig 15:**
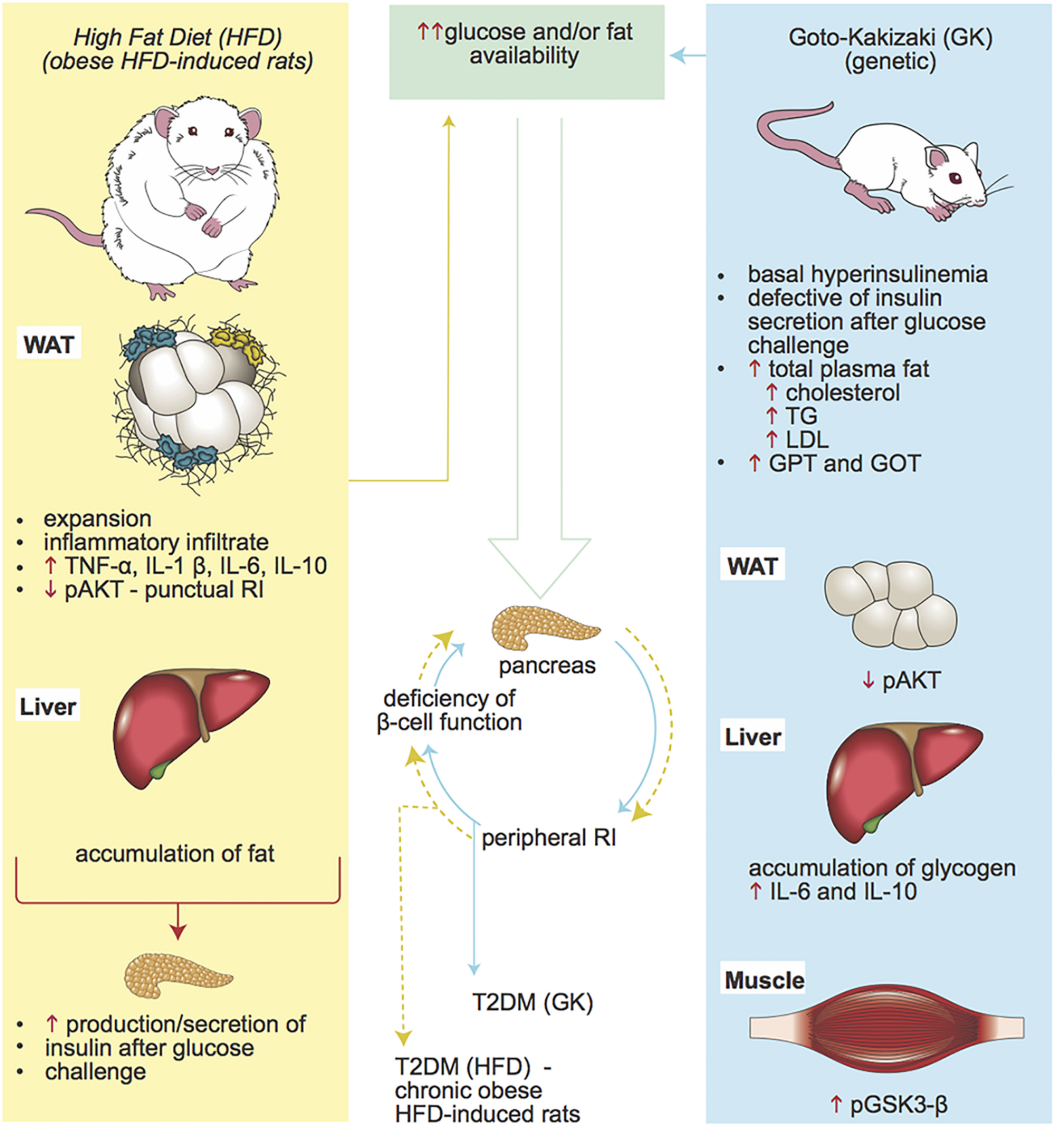
GK rats versus obese HFD induced rats. Two experimental models to study the development of T2DM.

Intrinsic versus extrinsic stimulus to the development T2DM was the main subject in this work. The choice of an experimental model is crucial to draw reliable conclusions and understand the correct pathophysiology of the disease, given the correct scenario. Herein, we showed two completely different approaches and tested their reliability as experimental models to study the development of T2DM. The genetic model, GK rats, presented all the typical hallmarks of this syndrome, which could be clearly observed after 2 months after birth. However, even before birth these animals already demonstrate loss of β cell mass and islet microangiopathy [12, 77–79], indicating that the main defect of this model is encountered in the insulin production. Consequently to the impairment in the capacity to secrete and produce insulin, the other characteristics follow up, such as hyperglycemia, increase in plasma fat and TG, liver inflammation, glucotoxicity and so on. Contrary to that, HFD induced a completely different scenario. In these animals, liver and muscle responded well to insulin stimulus, even though liver was found to have a great fat accumulation. However, we observed that HFD fed rats had lower insulin sensitivity and had to produce a greater amount of insulin to maintain normal glucose levels. This was mainly because the excessive fat accumulation in the WAT, which caused leukocyte infiltration and inflammation at this site. This inflammatory process was responsible to the impairment of insulin response in this tissue, as observed by decreased AKT phosphorylation. Our results showed that 8 weeks of HFD (60%) feeding caused insulin sensitivity impairment, but was not sufficient to induce T2DM in Wistar rats. We claim that longer period of HFD feeding would lead to pancreatic beta cell failure, decrease in insulin levels towards a glucose stimulus, hyperglycemia and finally the establishment of T2DM (Fig 15).

In sum, we can conclude that both experimental models are important tools to understand the different changes that intrinsic (genetics) and extrinsic factors (diet) might cause on the metabolism and physiology of the individuals. However, when GK rats and HFD fed rats (8 weeks) were compared, only GK rats were shown to be a reliable model to study the consequences of T2DM on the physiological systems, since HFD was unable to induce diabetes. Extrinsic factors can be regulated in order to avoid the progression of the disease and the genetic background is determinant in the development of T2DM, even in the absence of extrinsic factors influence. In order to draw the correct conclusion, studies have to consistently consider the differences in the development of T2DM and the factors that influence the progression of this disease.

## Supporting information

S1 Fig Percentage of total fat (A) and profile of fatty acids in the plasma of the animals (B-D). The determinations were performed by gas chromatography after lipid plasma extraction. Percentage of saturated fatty acids (B), monounsaturated fatty acids (C) and polyunsaturated fatty acids (D). Results are presented as mean ± S.E.M and n represents the number of animals used in each group. Studied groups: Control (n = 5); HFD (n = 5) and GK (n = 5). (*) p <0.05 *vs* control; (**) p <0.01 *vs* control; (##) indicates p <0.01 *vs* HFD; (###) p <0.001 *vs* HFD; (ϕϕ) indicates p <0.01 *vs* GK; (ϕϕϕ) indicates p <0.001 *vs* GK.
(TIFF)

S2 Fig Plasma adiponectin (A) and leptin (B) determination. Results are presented as mean ± S.E.M and n represents the number of animals used in each group. Studied groups: Control (n = 24); HFD (n = 20) and GK (n = 27).
(TIFF)

S3 Fig Hepatic enzymes: GPT (A) and GOT (B). Quantification was carried out in the animals’ plasma by enzymatic-colorimetric method. Results are presented as mean ± S.E.M and n represents the number of animals used in each group. Studied groups: Control (n = 24); HFD (n = 20) and GK (n = 31). (*) p <0.05 *vs* control; (**) p <0.01 *vs* control; (#) p <0.05 *vs* HFD; (###) p <0.001 *vs* HFD.
(TIFF)

S4 Fig Cytokines content in soleus muscle. Graphs present mean O.D. ± S.E.M of the bands and n represents the number of animals used in each group. Studied groups: Control (n = 4); HFD (n = 4) and GK (n = 4).
(TIFF)

S5 Fig Soleus muscle content of pAKT and AKT. (TIFF)

S6 Fig Soleus muscle content of pGSK-3β and pGSK-3β. (TIFF)

S7 Fig Liver content of pAKT and AKT. (TIFF)

S8 Fig Liver content of pGSK-3β and pGSK-3β. (TIFF)

S9 Fig Retroperitoneal white adipose tissue content of pAKT and AKT. (TIFF)

S10 Fig Retroperitoneal white adipose tissue content of pGSK-3β and pGSK-3β. (TIFF)

S11 Fig Cytokines content in liver. (TIFF)

S12 Fig Cytokines content in retroperitoneal white adipose tissue. (TIFF)
