# Supplementary figures and images for: Comparison of Goto-Kakizaki rats and high fat diet-induced obese rats: Are they reliable models to study Type 2 Diabetes mellitus?
Source: PLoS One. 2017 Dec 8;12(12):e0189622. doi: 10.1371/journal.pone.0189622 (PMC5722336; doi:10.1371/journal.pone.0189622)

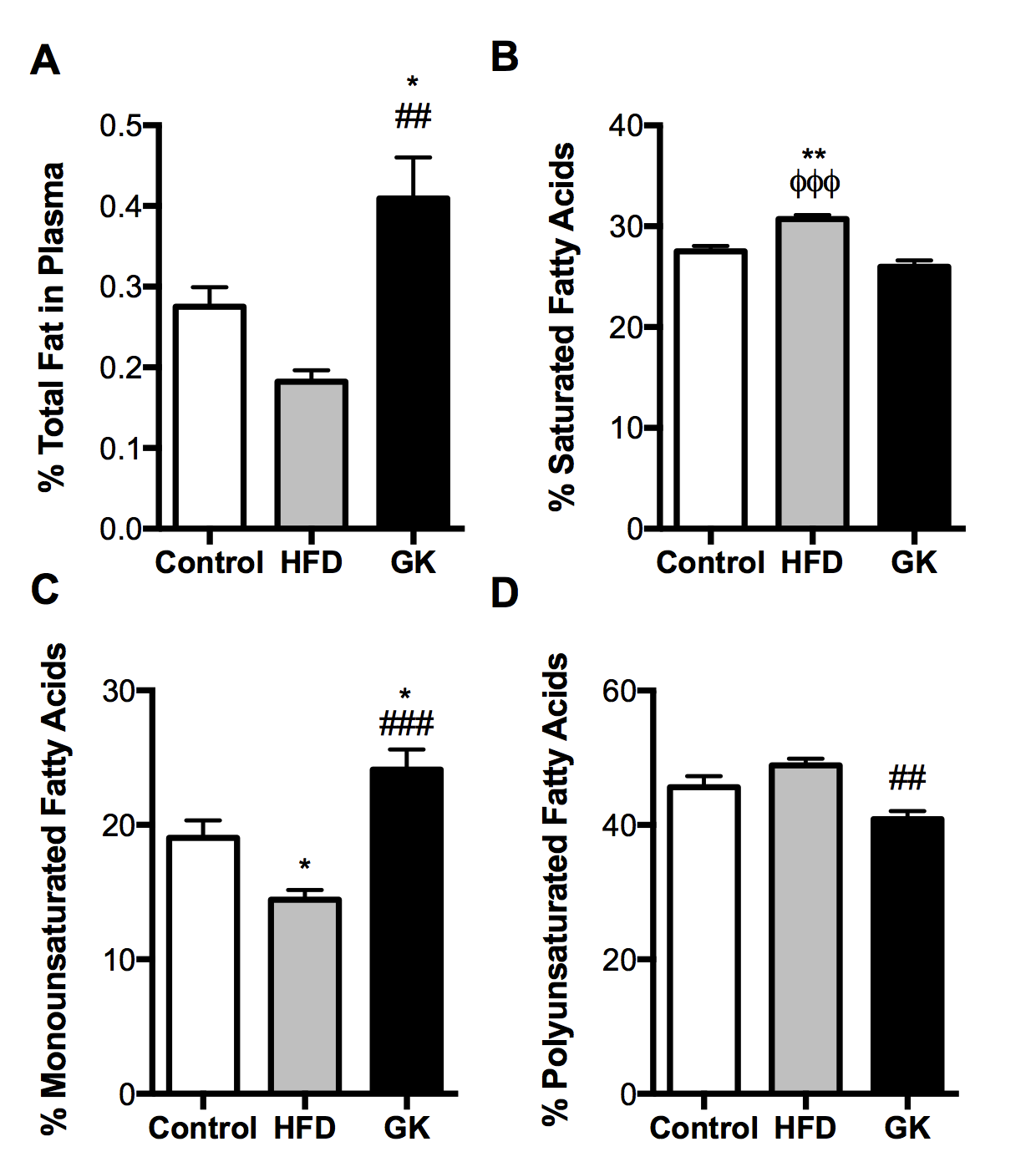

Supplement: S1 Fig — The determinations were performed by gas chromatography after lipid plasma extraction. Percentage of saturated fatty acids (B), monounsaturated fatty acids (C) and polyunsaturated fatty acids (D). Results are presented as mean ± S.E.M and n represents the number of animals used in each group. Studied groups: Control (n = 5); HFD (n = 5) and GK (n = 5). (*) p <0.05 vs control; (**) p <0.01 vs control; (##) indicates p <0.01 vs HFD; (###) p <0.001 vs HFD; (ϕϕ) indicates p <0.01 vs GK; (ϕϕϕ) indicates p <0.001 vs GK. (TIFF) [file pone.0189622.s001.tiff]

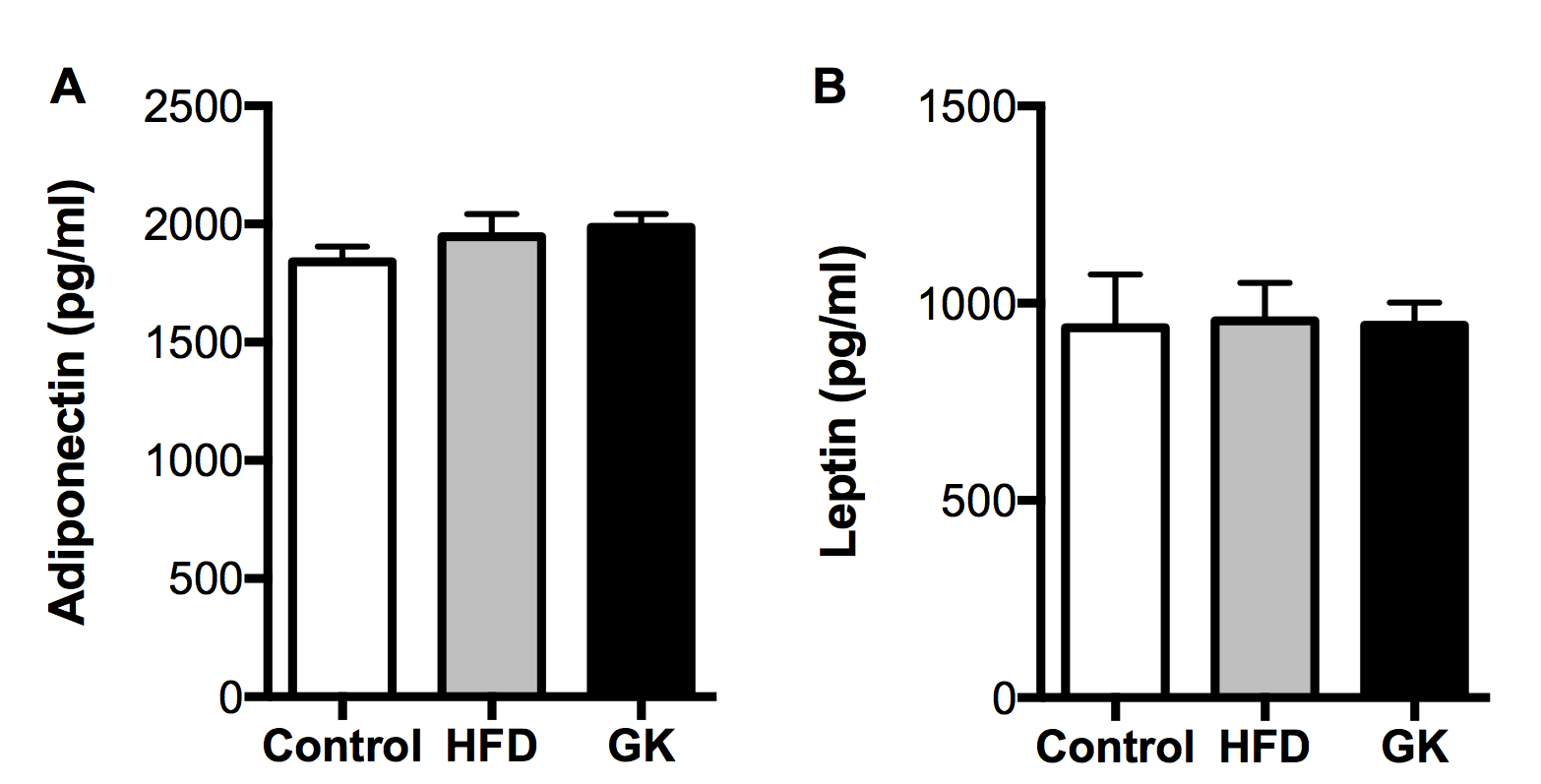

Supplement: S2 Fig — Results are presented as mean ± S.E.M and n represents the number of animals used in each group. Studied groups: Control (n = 24); HFD (n = 20) and GK (n = 27). (TIFF) [file pone.0189622.s002.tiff]

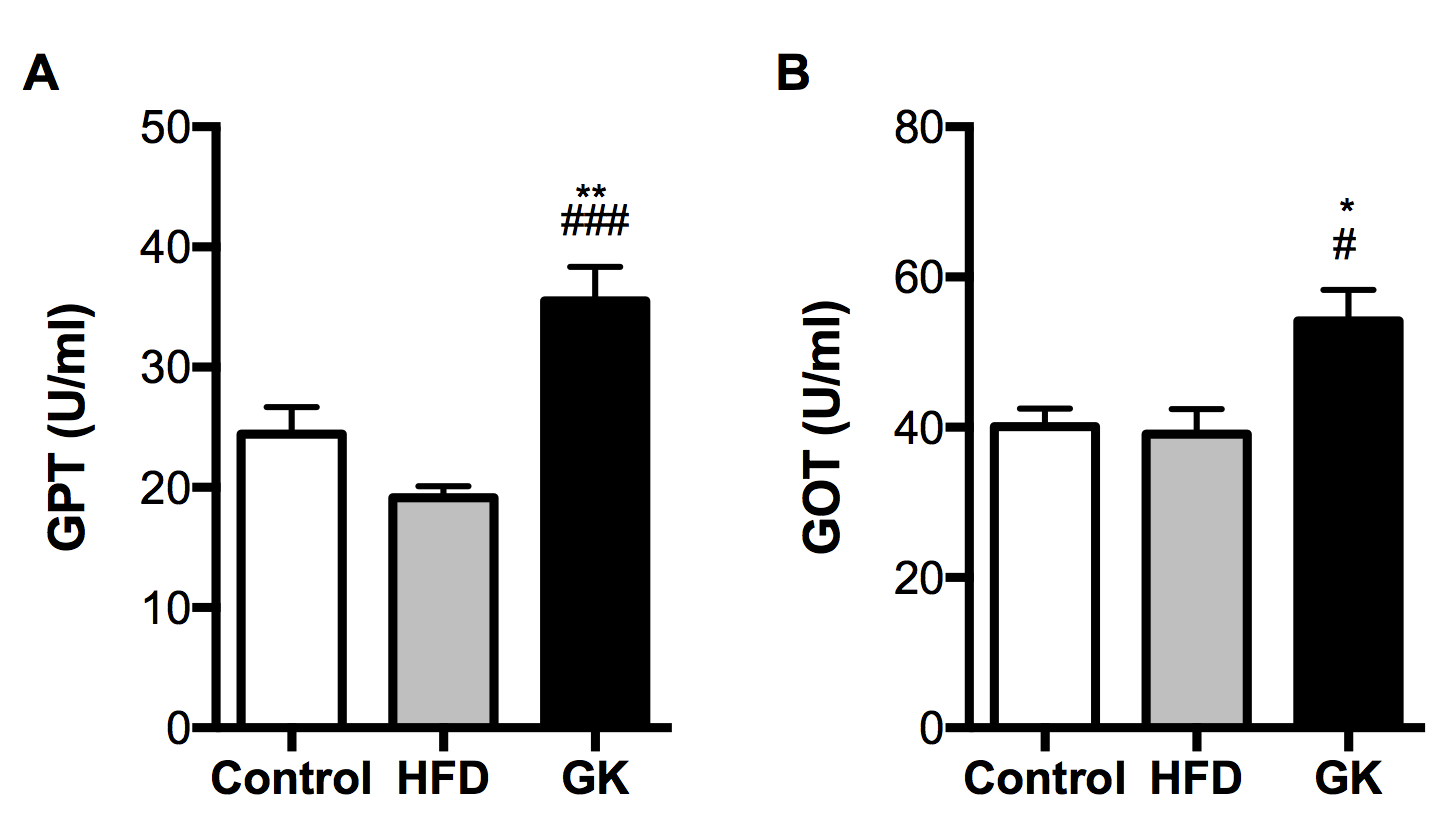

Supplement: S3 Fig — Quantification was carried out in the animals’ plasma by enzymatic-colorimetric method. Results are presented as mean ± S.E.M and n represents the number of animals used in each group. Studied groups: Control (n = 24); HFD (n = 20) and GK (n = 31). (*) p <0.05 vs control; (**) p <0.01 vs control; (#) p <0.05 vs HFD; (###) p <0.001 vs HFD. (TIFF) [file pone.0189622.s003.tiff]

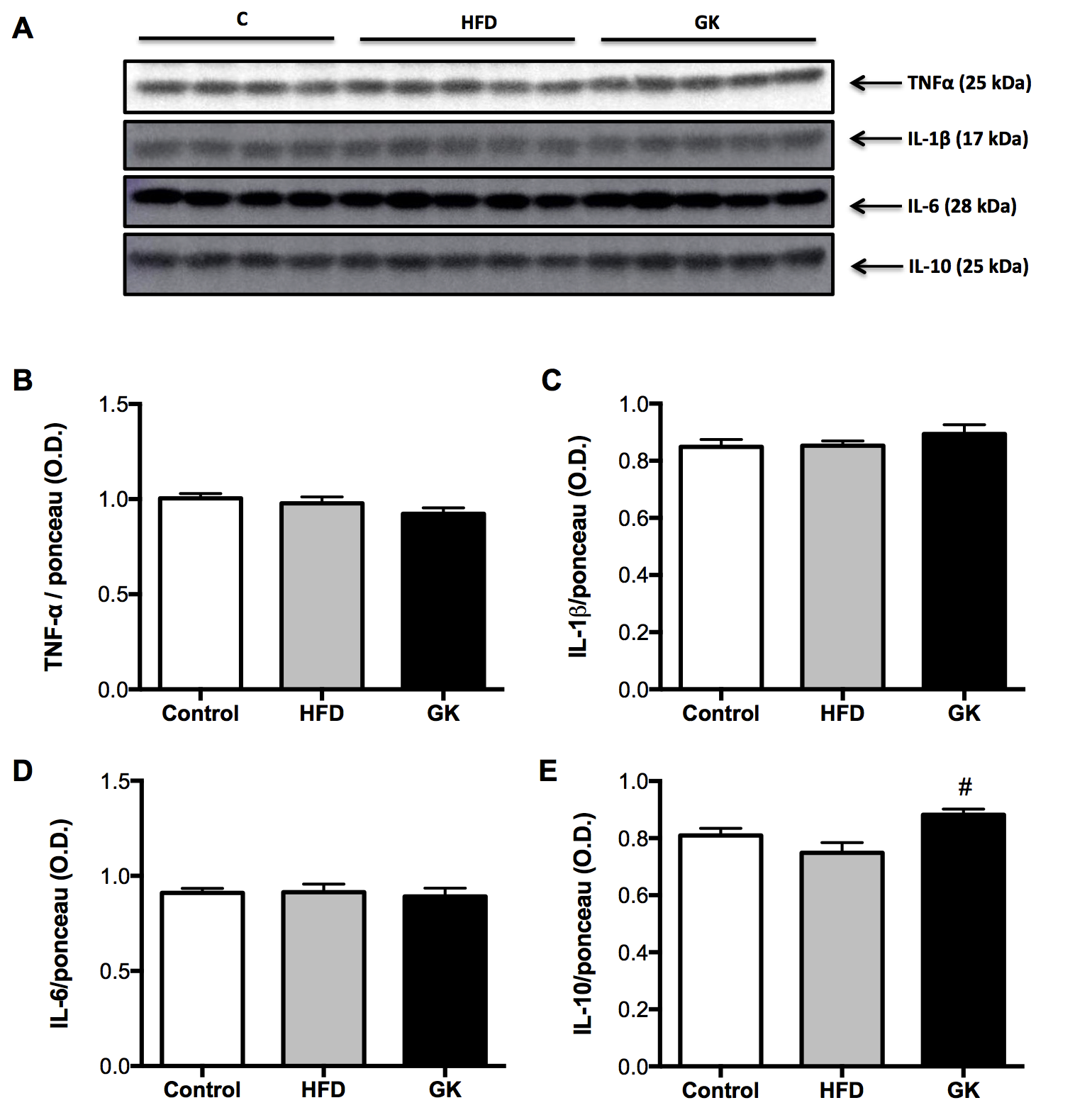

Supplement: S4 Fig — Graphs present mean O.D. ± S.E.M of the bands and n represents the number of animals used in each group. Studied groups: Control (n = 4); HFD (n = 4) and GK (n = 4). (TIFF) [file pone.0189622.s004.tiff]

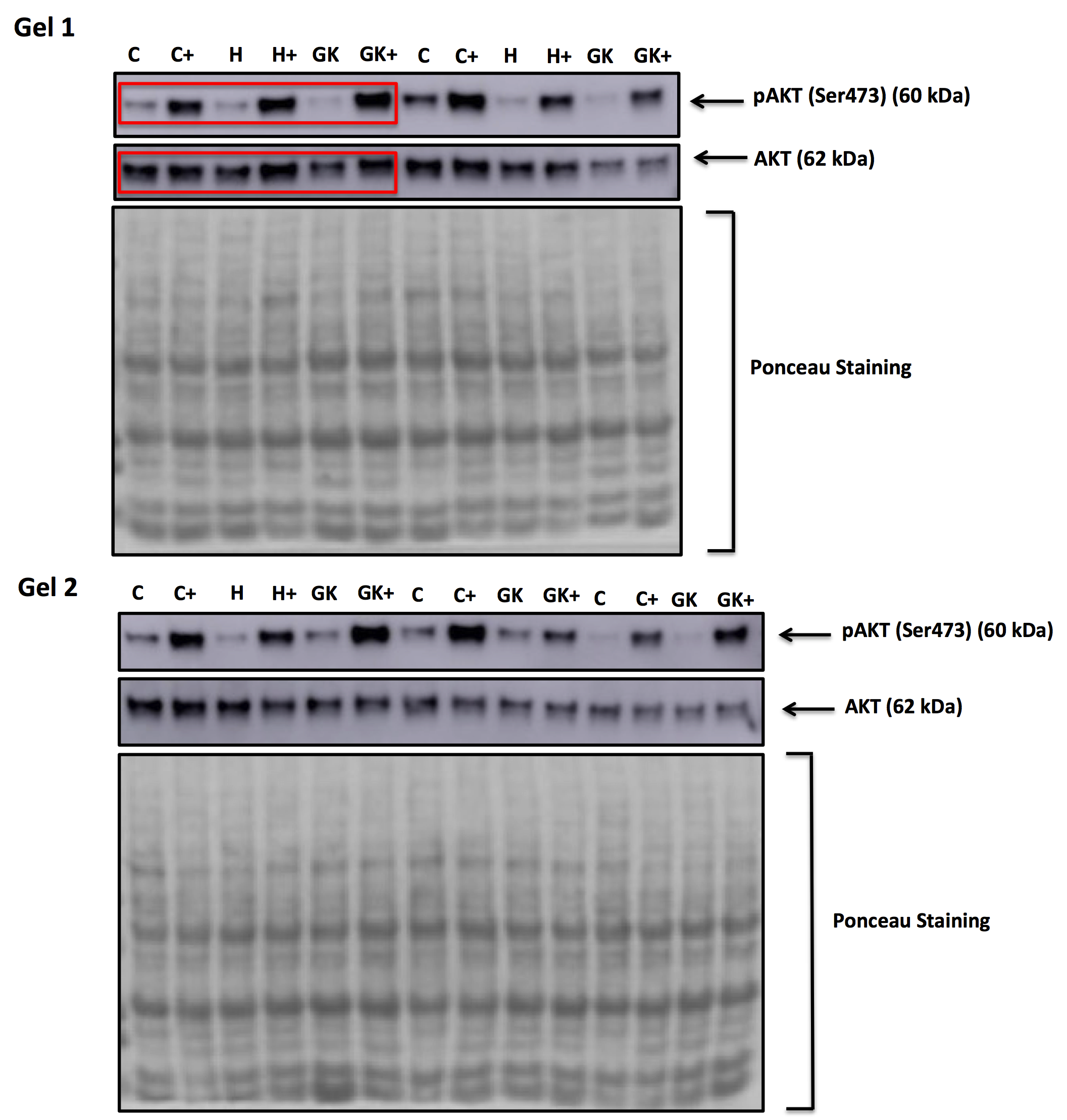

Supplement: S5 Fig — (TIFF) [file pone.0189622.s005.tiff]

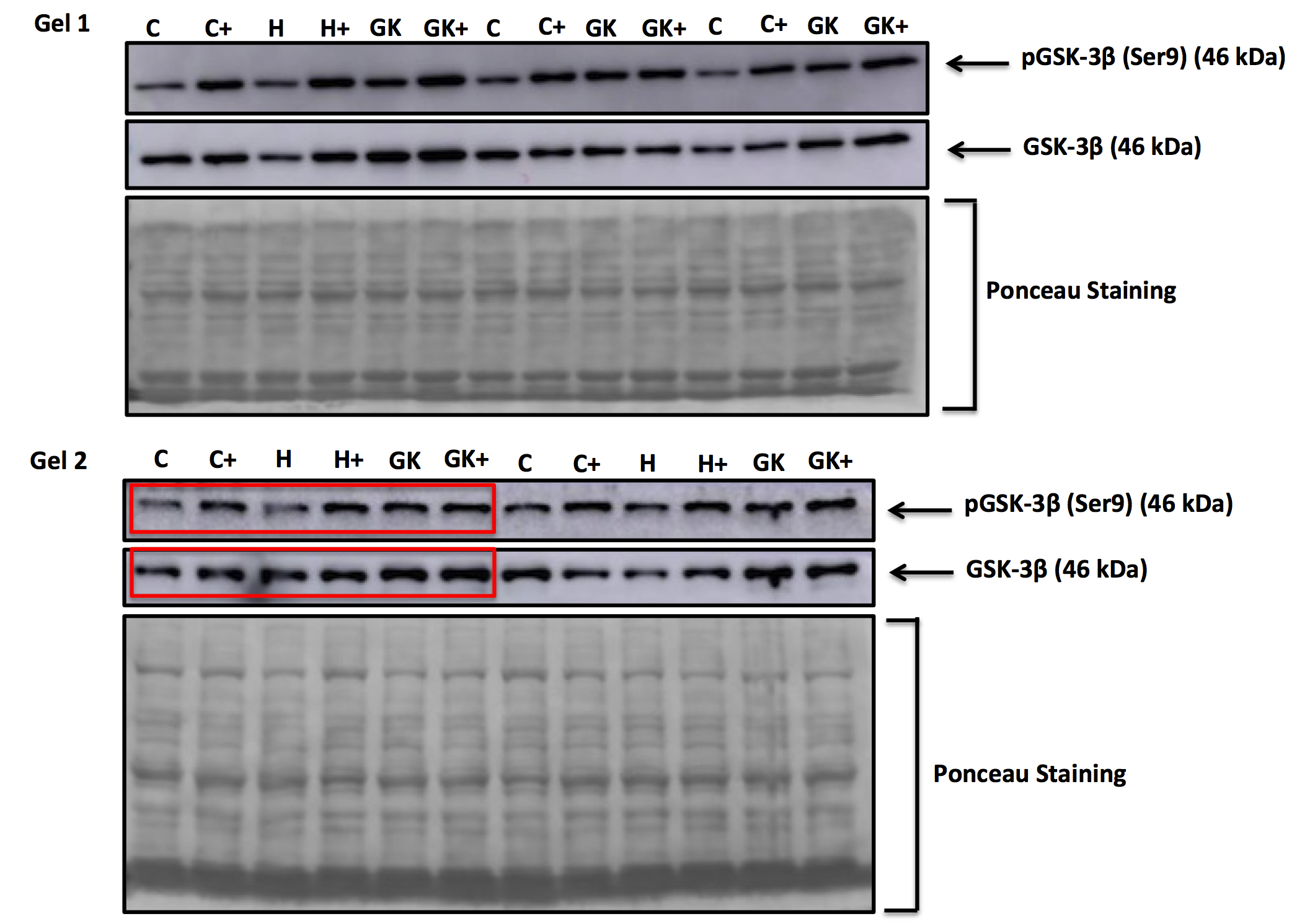

Supplement: S6 Fig — (TIFF) [file pone.0189622.s006.tiff]

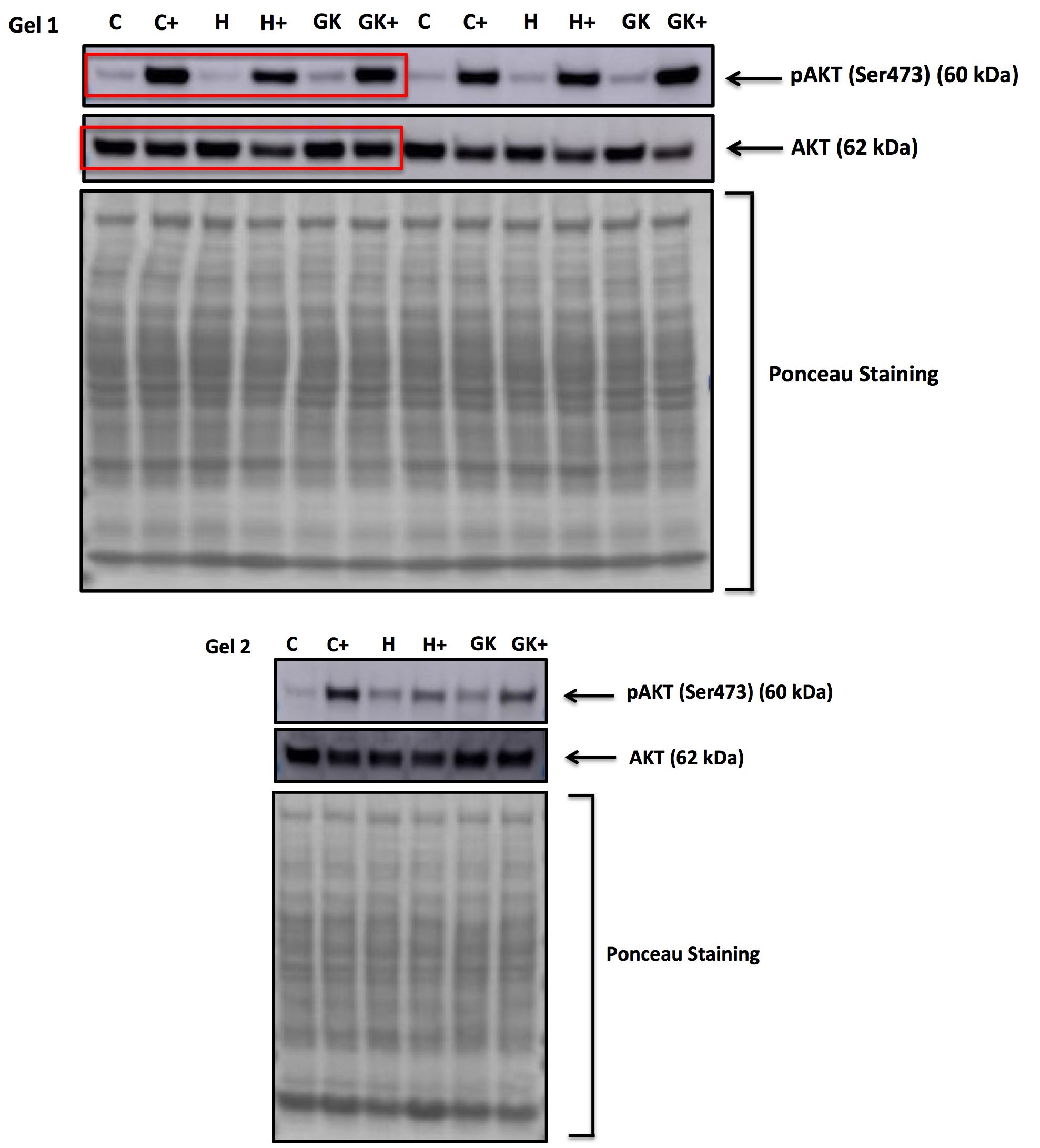

Supplement: S7 Fig — (TIFF) [file pone.0189622.s007.tiff]

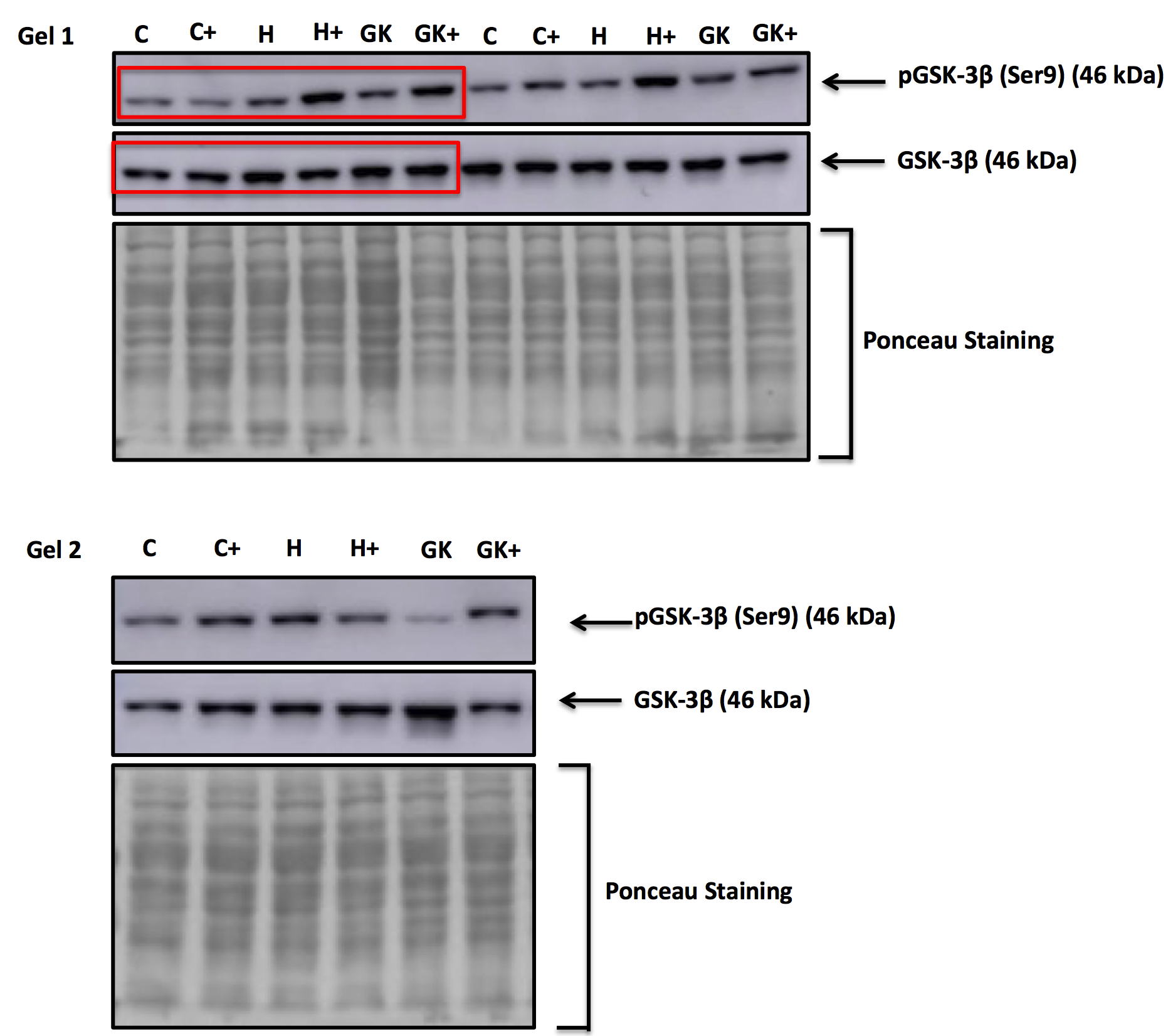

Supplement: S8 Fig — (TIFF) [file pone.0189622.s008.tiff]

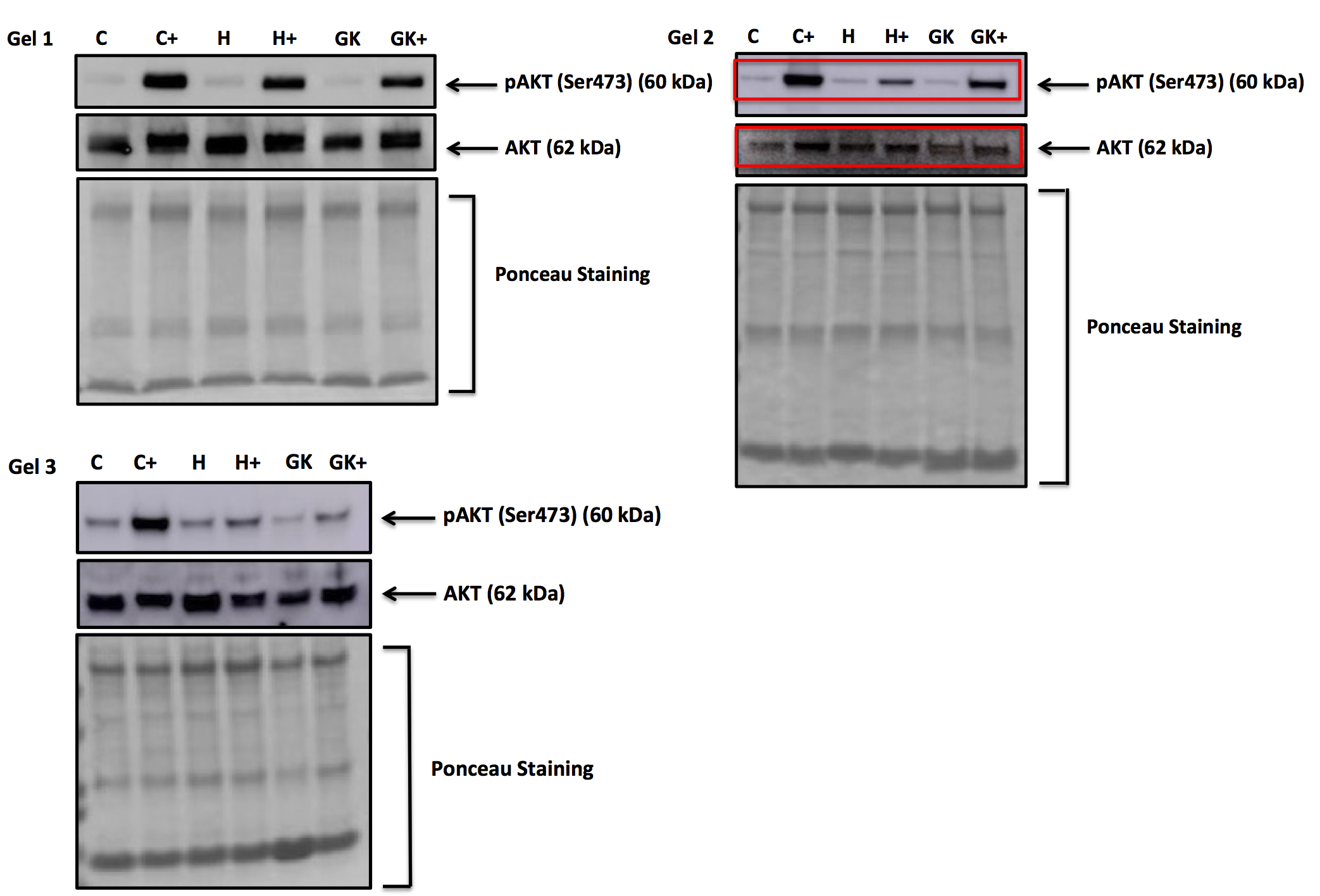

Supplement: S9 Fig — (TIFF) [file pone.0189622.s009.tiff]

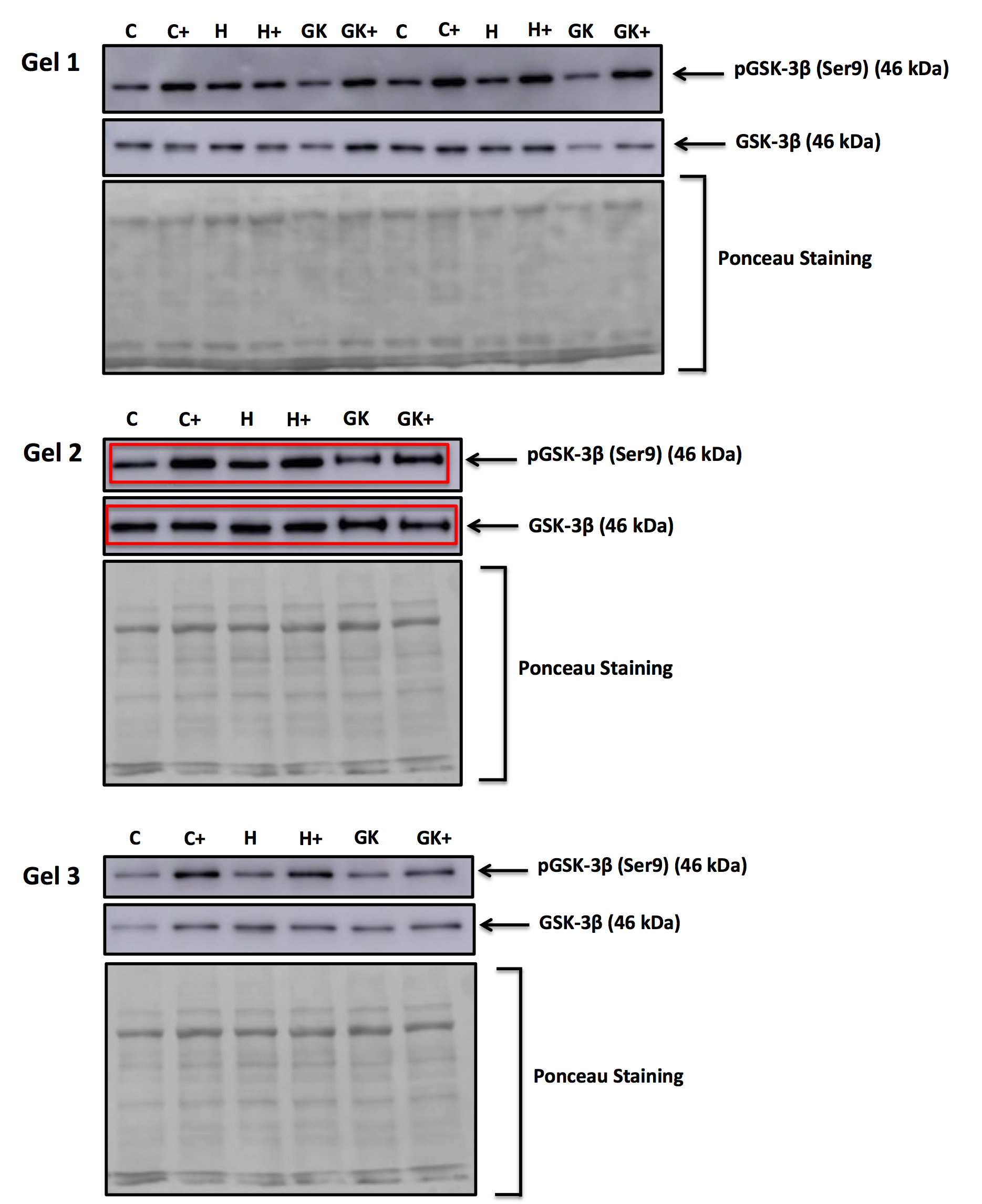

Supplement: S10 Fig — (TIFF) [file pone.0189622.s010.tiff]

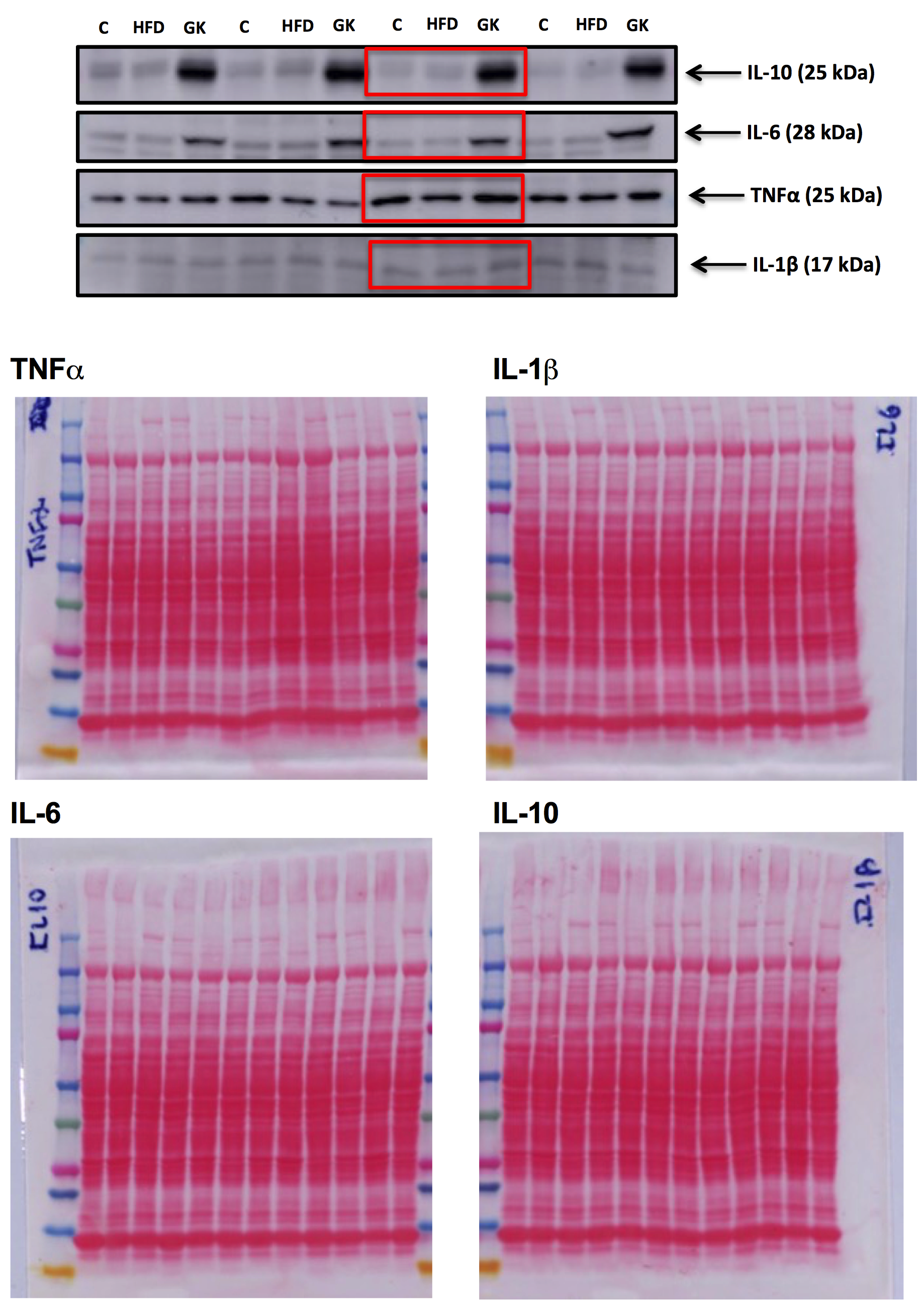

Supplement: S11 Fig — (TIFF) [file pone.0189622.s011.tiff]

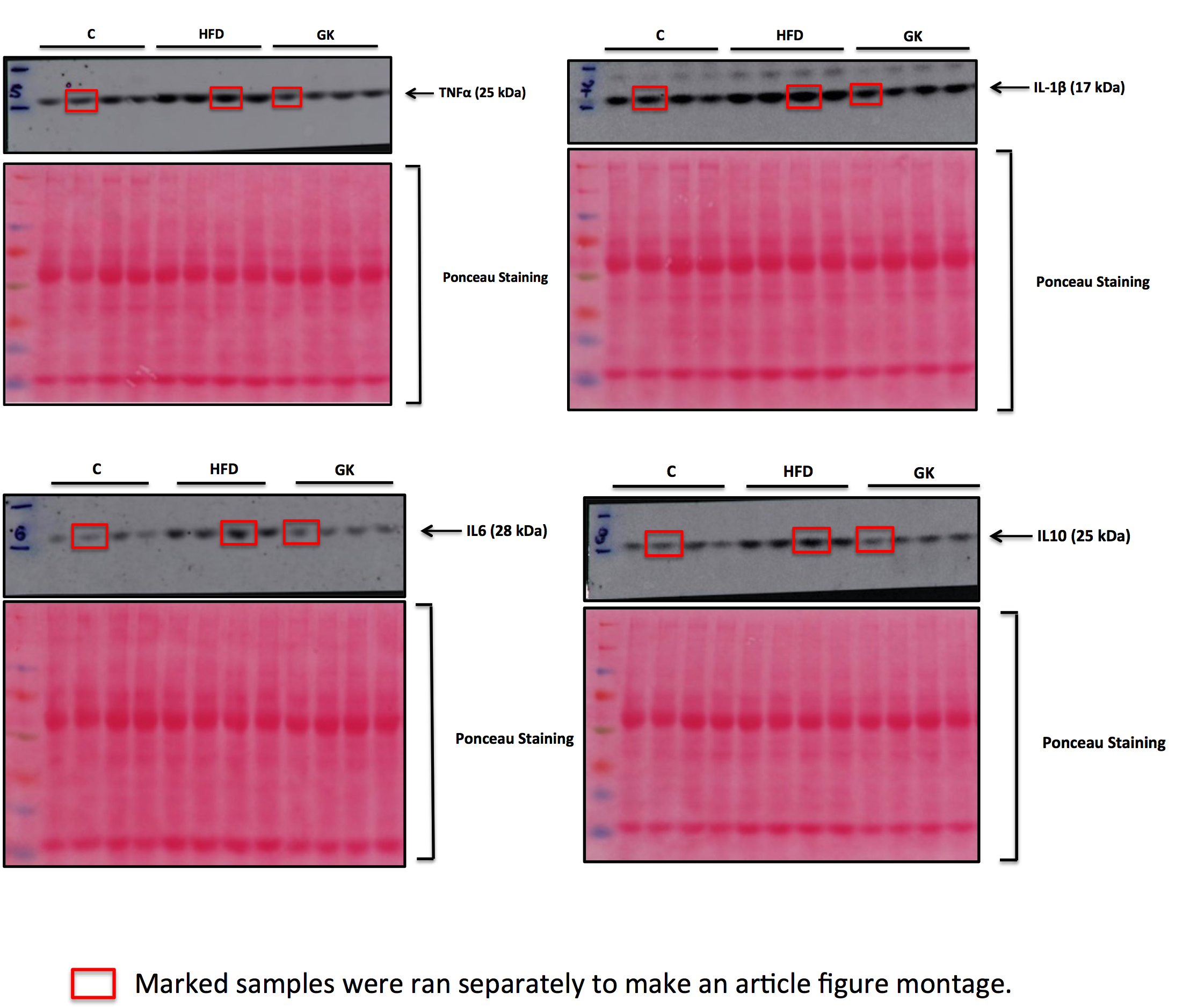

Supplement: S12 Fig — (TIFF) [file pone.0189622.s012.tiff]
